# SEC23A confers ER stress resistance in gastric cancer by forming the ER stress-SEC23A-autophagy negative feedback loop

**DOI:** 10.1186/s13046-023-02807-w

**Published:** 2023-09-05

**Authors:** Quan Cheng, Kanghui Liu, Jian Xiao, Kuan Shen, Yuanhang Wang, Xinyi Zhou, Jiawei Wang, Zekuan Xu, Li Yang

**Affiliations:** 1 Department of General Surgery, the First Affiliated Hospital of Nanjing Medical University, 300 Guangzhou Road, Nanjing, 210029 Jiangsu Province China; 2 Department of General Surgery, Liyang People’s Hospital, Liyang Branch Hospital of Jiangsu Province Hospital, Liyang, Jiangsu Province China

**Keywords:** 5-FU, Apoptosis, Autophagy, ER stress, Gastric cancer, SEC23A

## Abstract

**Background:**

Sec23 homolog A (SEC23A), a core component of coat protein complex II (COPII), has been reported to be involved in several cancers. However, the role of SEC23A in gastric cancer remains unclear.

**Methods:**

The expression of SEC23A in gastric cancer was analyzed by using qRT-PCR, western blotting and IHC staining. The role of SEC23A in ER stress resistance was explored by functional experiments in vitro and vivo. The occupation of STAT3 on the SEC23A promoter region was verified by luciferase reporter plasmids and CHIP assay. The interaction between SEC23A and ANXA2 was identified by Co-IP and mass spectrometry analysis.

**Results:**

We demonstrated that SEC23A was upregulated in gastric cancer and predicted poor prognosis in patients with gastric cancer. Mechanistically, SEC23A was transcriptional upregulated by ER stress-induced pY705-STAT3. Highly expressed SEC23A promoted autophagy by regulating the cellular localization of ANXA2. The SEC23A-ANXA2-autophay axis, in turn, protected gastric cancer cells from ER stress-induced apoptosis. Furthermore, we identified SEC23A attenuated 5-FU therapeutic effectiveness in gastric cancer cells through autophagy-mediated ER stress relief.

**Conclusion:**

We reveal an ER stress-SEC23A-autophagy negative feedback loop that enhances the ability of gastric cancer cells to resist the adverse survival environments. These results identify SEC23A as a promising molecular target for potential therapeutic intervention and prognostic prediction in patients with gastric cancer.

**Supplementary Information:**

The online version contains supplementary material available at 10.1186/s13046-023-02807-w.

## Background

Gastric cancer (GC) is the fifth most prevalent malignancy and the fourth leading cause of cancer-related mortality worldwide [1, 2]. It is critical to investigate the primary causes and underlying mechanisms of poor prognosis in GC patients. Chemotherapy continues to be the principal treatment for advanced GC. Chemotherapy resistance and unavoidable toxicity drastically limit the therapeutic effects of the treatment, resulting in a dismal prognosis for patients with GC [3]. The majority of GC patients usually have a poor prognosis due to late diagnosis and inadequate response to available treatments [4]. Therefore, it is essential to explore the mechanisms of GC progression and chemotherapy resistance in order to identify effective therapeutic targets and improve the prognosis of GC patients.

Endoplasmic reticulum (ER) stress caused by intrinsic and extrinsic variables and adverse survival environments, such as increased metabolic demand, reactive oxygen species (ROS) overproduction, and cytotoxic medicines, which tumor cells frequently encounter, affects the function and survival of tumor cells [5]. The unfolded protein response (UPR) is the adaptive mechanism that restores ER homeostasis through a number of pathways, including transcriptional reprogramming and mRNA decay, global translational absorption, and recycling of misfolded proteins and cellular materials through autophagy induction [5, 6]. Activation of the UPR promotes cell adaptation to stress and survival by restoring ER homeostasis. However, if ER stress persists and homeostasis cannot be restored, the UPR triggers cell death in cells that are beyond repair [6, 7]. Multiple unfavorable survival environments for tumor growth have the potential to cause ER stress and then activate the UPR, which shields cancer cells from ER stress-induced apoptosis and promotes tumor progression and therapy resistance [8–10]. Examining the mechanisms by which GC cells evade apoptosis when exposed to ER stress and identifying prospective therapeutic targets for ER stress-induced cell death are crucial for impeding tumor progression and enhancing chemotherapy efficacy.

Macroautophagy/autophagy is a highly conserved cellular process that is rigorously governed by a series of signaling pathways [11]. Autophagy recycles cell components and supplies macromolecular precursors by delivering cell materials to lysosomes for degradation via double-membrane vesicles called autophagosomes [12]. Autophagy can be induced over the basal level of activity by many cellular stresses, including ER stress [13]. As a primarily pro-survival process, the autophagy eliminates misfolded proteins to recover ER homeostasis under conditions of ER stress [14, 15]. During chemotherapy, cytoprotective autophagy suppression has been shown to increase ER stress-induced cell death in cancer cells [16, 17].

Sec23 homolog A (SEC23A) is a core component of coat protein complex II (COPII), which helps transport proteins and lipids from the endoplasmic reticulum to the Golgi apparatus [18]. Functionally, SEC23A promotes vesicle transport, regulates protein secretion, and controls membrane protein trafficking [19–22]. Previous studies have revealed that SEC23A regulates autophagy and mediates ER stress-induced unfolded protein response (UPR) recovery by eliminating excess ER in a process called ER-phagy [23]. SEC23A overexpression has been linked to a poor prognosis in bladder and gastric cancers [24]. Recent studies have demonstrated that SEC23A inhibits melanoma metastasis via autocrine activation of autophagy in extravasated tumor cells and inhibition of the MAPK signaling pathway [25, 26]. However, the functions and underlying mechanisms by which SEC23A is involved in GC progression have not been investigated.

In the present study, we reported that SEC23A is highly expressed in GC cells under ER stress, which is important for ER stress relief and avoidance of ER stress-induced apoptosis via the autophagy induction. ER stress, SEC23A and autophagy form a negative feedback loop that maintain GC cells survival advantage during ER stress. The ER stress-induced activation of SEC23A transcription requires activation of the JAK2–STAT3 (Janus kinase 2–signal transducer transducer and activator of transcription 3) signaling pathway to phosphorylate STAT3 at Tyr705, which binds to the SEC23A promoter region more frequently during ER stress. In addition, we demonstrated that SEC23A facilitates Annexin A2 (ANXA2) plasma membrane trafficking, resulting in increased autophagic flux in GC cells. Moreover, SEC23A suppression significantly enhanced 5-fluorouracil (5-FU) efficacy for GC cells in vitro and in vivo by inhibiting autophagy and relieving ER stress. Due to its cytoprotective function during ER stress, SEC23A may represent a promising molecular target whose inhibition could be used to inhibit GC development and improve chemotherapeutic efficacy.

## Methods

### Patient tissue samples

A total of 82 paired GC and adjacent normal tissues were obtained at the First Affiliated Hospital of Nanjing Medical University for comparisons of SEC23A expression between paired tumor and paratumor tissues. Of these, 30 pairs of tissues were used for immunohistochemical staining, 12 pairs for western blotting, and 82 pairs were used for qRT-PCR. None of these patients have received chemotherapy or radiotherapy. The research has been endorsed by the Medical Ethics Committee of the First Affiliated Hospital of Nanjing Medical University.

### Cell culture and transfection

MKN45 and HGC27 human GC cell lines were purchased from Cell Bank of the Chinese Academy of Sciences (Shanghai, China) and were cultured in RPMI-1640 (Wisent, 350–000-CL) supplemented with 10% fetal bovine serum (FBS) (Gibco, 10270–106) in an incubator supplemented with 5% CO_2_ and operating at 37 °C.

To establish stable SEC23A-knockdown and SEC23A-overexpressing GC cells, lentivirus vectors containing short-hairpin RNA (shRNA) sequences against SEC23A (shSEC23A-1 and shSEC23A-2), SEC23A sequence plasmid (oeSEC23A) and their control vectors (shNC and vector) were obtained from GEMA (Shanghai, China).

For the silencing of STAT3, ATF6, ATF4, CHOP, XBP1 and CREB3L2, MKN45 and HGC27 cells were transiently transfected with small interfering RNAs (siRNAs) (GEMA, Shanghai, China) targeting these mRNAs and their negative controls by using the Lipofectamine 3000 Reagent (Invitrogen, L3000015) according to the protocol.

All the sequences are listed in Supplementary Table S1.

### Quantitative RT-PCR

Total RNA was extracted from cultured cells as described in a previous study [27]. The cDNA synthesis and quantitative PCR assays were performed using HiScript III All-in-one RT SuperMix Perfect for qPCR (Vazyme, R333-01) and AceQ qPCR SYBR Green Master Mix (Vazyme, Q141-02) according to the kit protocols. All samples were measured with QuantStudio 7 system (Thermo Fisher Scientific, USA) in 96-well plate. The relative expression levels were standardized to the internal control GAPDH using the ?Ct method.

The primers used for qPCR were listed in Supplementary Table S2.

### Western blotting

Cell protein was extracted with RIPA Lysis Buffer (Beyotime, P0013B) and the concentration was detected by Enhanced BCA Protein Assay Kit (Beyotime, P0010). Western blotting was performed as described previously [27]. The results were measured by ImageJ software.

The antibodies were used for western blotting are listed in Supplementary Table S3.

### Animal studies

The animal procedures were approved by the Committee on the Ethics of Animal Experiments of Nanjing Medical University. All the mice were purchased from the Animal Center of Nanjing Medical University. For the subcutaneous tumor formation models, five mice per group, 2 × 10^6^ shNC and shSEC23A MKN45 cells were diluted with PBS to 100 µl and then injected into the axilla of the forelimbs of 4- to 5-week-old nude mice. 12 days after inoculation, mice were intraperitoneally injected with drugs respectively (100 µl/d PBS for the control group, 0.5 mg/kg/d TM or 0.5 mg/kg/d 5-FU for the treatment groups) every four days. The tumor volume was monitored every 2 days postinjection and calculated with the following formula: V = p /6 (Length × Width^2^). At the 4th week postinoculation, animals were sacrificed and tumors were collected and weighed.

### Bioinformatics and statistics

Expression differences and survival analyses of SEC23A in TCGA dataset were obtained from GEPIA2 database. Survival analyses in GEO dataset and 82 GC patients from our center, univariate and multivariate Cox regression analysis, GSEA, and Spearman correlation analysis were performed with RStudio (1.4.1717).

All experiments were preformed three times independently and the results are reported as mean ± SD.Comparisons of SEC23A expression between paired tumor and paratumor tissues were performed by paired Student’s t-test. The relationship between SEC23A expression and clinicopathological characteristics was analyzed with chi-square test. Differences between experimental and control groups were analyzed with unpaired Student’s t-test for parametric tests and Wilcoxon signed-rank test for nonparametric tests. Values of *p* < 0.05 were considered statistically significant. *p < 0.05; **p < 0.01; ***p < 0.001; ****p < 0.0001.

## Results

### SEC23A was upregulated in GC and predicted a poor prognosis in patients with GC

**Fig. 1:**
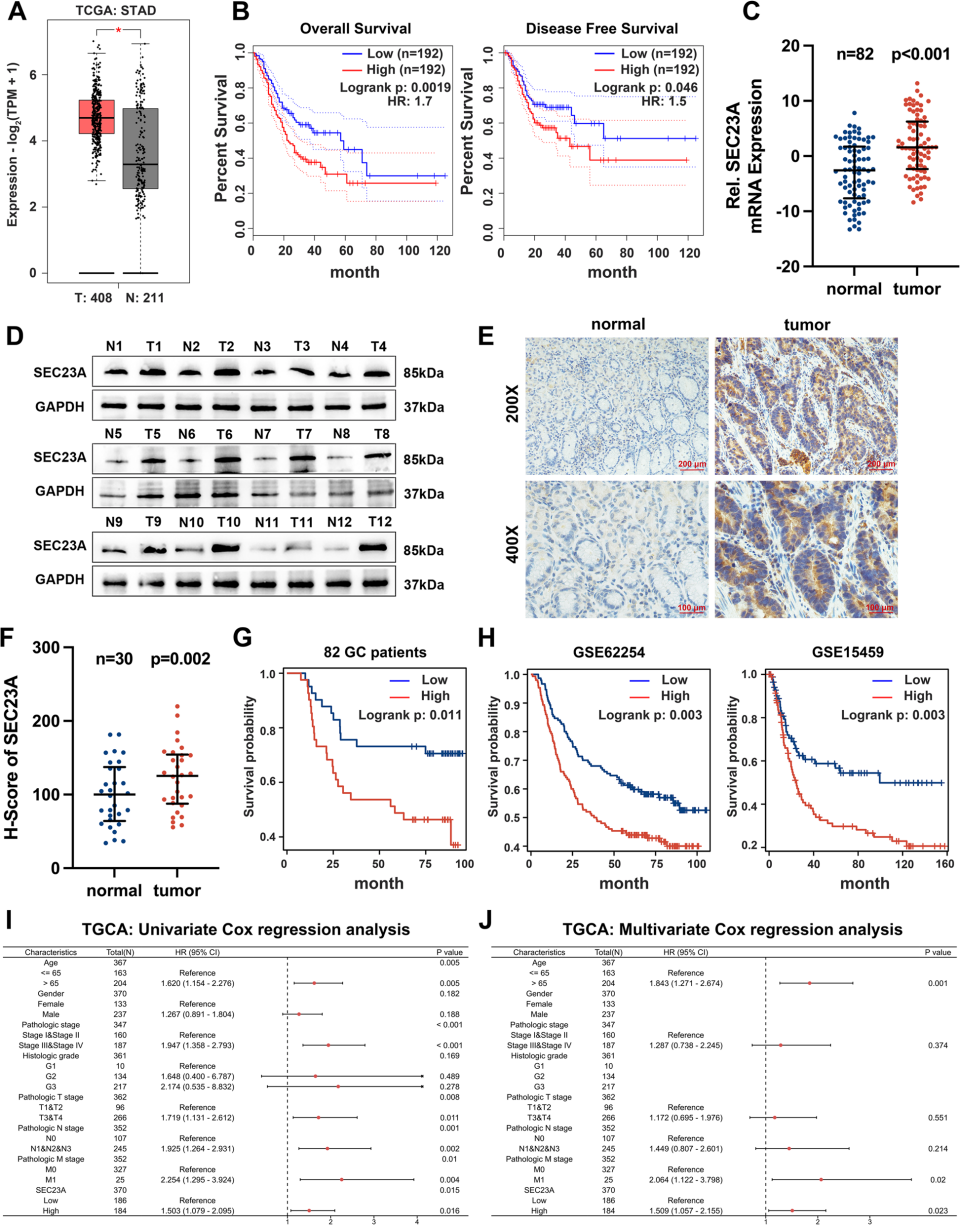
SEC23A was overexpressed in GC and indicated poor prognosis. **A **and** B** Expression and survival analysis of SEC23A in TCGA and GTEx dataset using GEPIA2 database. **C** SEC23A expression analysis using qRT-PCR in 82 paired GC and adjacent normal tissues. **D** SEC23A expression analysis using western blotting in 12 paired GC and adjacent normal tissues. **E **and** F** IHC staining images (**E**) and the H-Score (**F**) of SEC23A expression in 30 GC tissues and matched normal tissues. **G** Survival analysis of SEC23A using log-rank test in 82 GC patients. **H** Survival analysis of SEC23A using log-rank test in GSE62254 and GSE15459 datasets. **I **and** J** Univariate (**I**) and multivariate (**J**) Cox regression analysis of SEC23A expression in GC with TCGA dataset

According to the TCGA and GTEx datasets, we found that SEC23A was highly expressed in GC tissue (Fig. 1A). In addition, the GEPIA2 database revealed that GC patients with a higher SEC23A expression exhibited shortened overall survival and disease-free survival (Fig. 1B). These results suggested that SEC23A may take an essential part in the development of GC. According to qRT-PCR and western blot data, SEC23A was considerably upregulated in GC tissues when compared to adjacent normal tissues (Fig. 1C and D). IHC staining and the H-Score also confirmed the upregulation of SEC23A in GC tissues (Fig. 1E and F). The clinicopathologic and survival information for the 82 GC patients from our center showed that high SEC23A expression was associated with more advanced tumors and worse prognosis (Table 1 and Fig. 1G). Consistent survival analysis results were obtained from the GSE62254 and GSE15459 datasets (Fig. 1H). Univariate and multivariate Cox regression analyses with the TCGA dataset revealed high SEC23A expression was an independent indicator for predicting the overall survival of GC patients (Fig. 1I and J). Collectively, these results demonstrated that SEC23A was highly expressed in GC tissues and was associated with a poor prognosis.

**Table 1:** Relationships between SEC23A expression and the clinicopathologic characteristics in 82 gastric cancer patients

Characteristics	Group	Case	SEC23A expression		*p*-value
			Low	High	
Age	< 60	40	21 (25.6%)	19(23.2%)	0.659
	≥ 60	42	20 (24.4%)	22(26.8%)	
Gender	Male	32	19 (23.2%)	13 (15.9%)	0.174
	Female	50	22 (26.8%)	28 (34.1%)	
Tumor size	< 4 cm	53	34 (41.5%)	19 (23.2%)	** < 0.001**
	≥ 4 cm	29	7 (8.5%)	22 (26.8%)	
Tumor differentiation	Well + moderate	54	22 (26.8%)	32 (39%)	**0.020**
	Poor	28	19 (23.2%)	9 (11%)	
Tumor Invasion	T1-T2	36	25 (30.5%)	11 (13.4%)	**0.002**
	T3-T4	46	16 (19.5%)	30 (36.6%)	
Lymph node metastasis	Negative	34	20 (24.4%)	14 (17.1%)	0.179
	Positive	48	21 (25.6%)	27 (32.9%)	
TNM stage	I–II	39	24 (29.3%)	15 (18.3%)	**0.047**
	III–IV	43	17 (20.7%)	26 (31.7%)	

### SEC23A was induced in the condition of ER stress

**Fig. 2:**
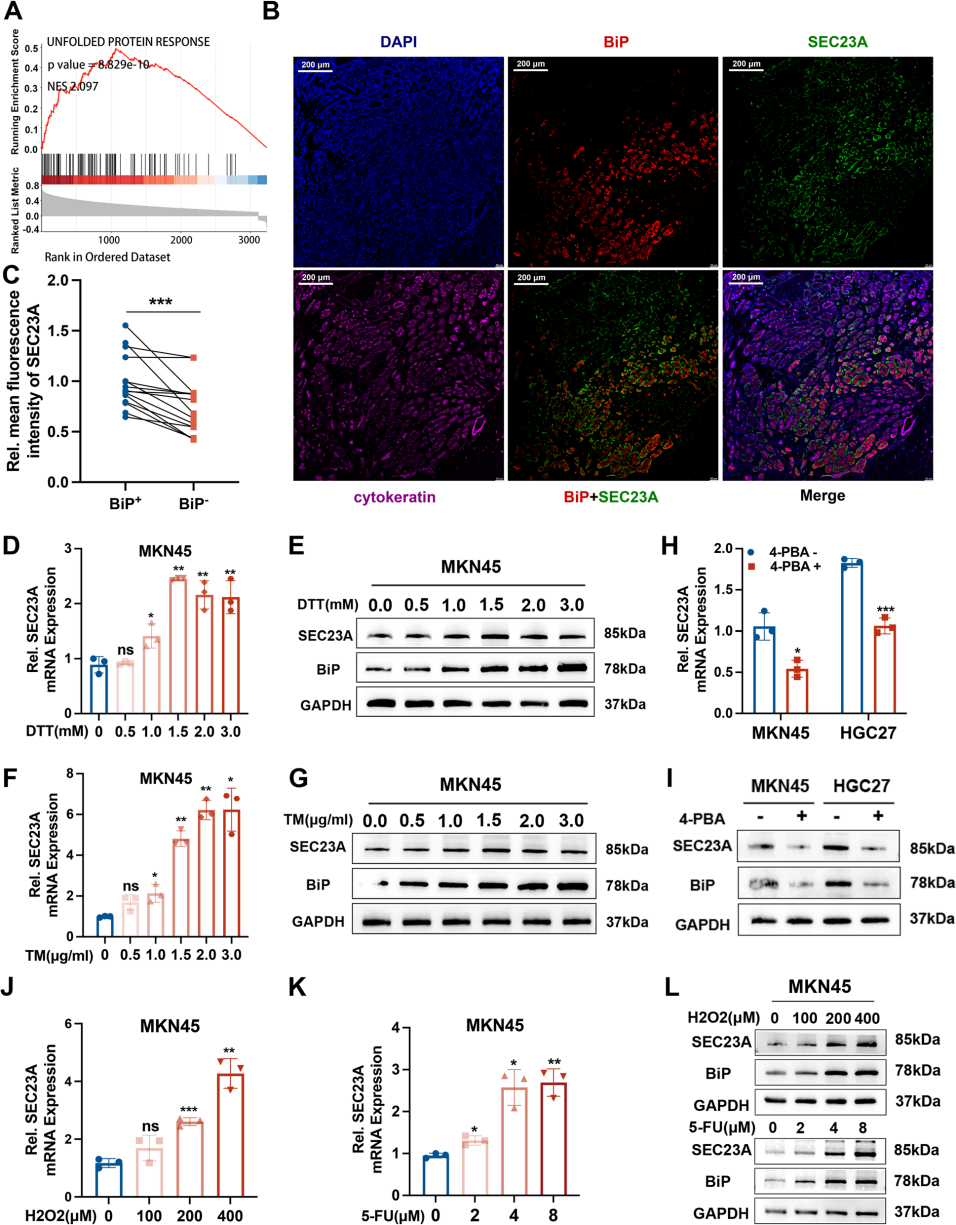
SEC23A was upregulated in GC cells during ER stress. **A** GSEA analysis of SEC23A using TCGA STAD mRNA expression data. **B** Representative images of immunofluorescence co-staining SEC23A, BiP and cytokeratin in 15 GC tissues. **C** Fluorescence intensity quantitative analysis of SEC23A expression in BiP^+^ and BiP^-^ areas of human GC tissues (*n* = 15). **D **and** E** qRT-PCR (**D**) and western blotting (**E**) analysis of SEC23A expression in MKN45 cells under concentration gradient treatments of DTT for 12 h. **F **and** G** qRT-PCR (**F**) and western blotting (**G**) analysis of SEC23A expression in MKN45 cells under concentration gradient treatments of TM for 12 h. **H **and** I** MKN45 cells were treated with 5 mM 4-PBA for 12 h, SEC23A expression was detected by qRT-PCR (**H**) and western blotting (**I**). **J** qRT-PCR analysis of SEC23A expression in MKN45 cells under concentration gradient treatments of H_2_O_2_ for 24 h. **K** qRT-PCR analysis of SEC23A expression in MKN45 cells under concentration gradient treatments of 5-FU for 24 h. **L** Western blotting analysis of SEC23A expression in MKN45 cells under concentration gradient treatments of H_2_O_2_ and 5-FU respectively for 24 h

To develop a basic understanding of the roles and mechanisms of SEC23A on GC, we conducted GSEA using TCGA dataset. Results indicated a significant correlation between SEC23A and UPR (Fig. 2A). Since UPR is the symbolic pathway of ER stress, we initially performed immunofluorescence staining of SEC23A in human GC tissues with GRP78/BiP, an ER stress marker in the UPR pathway [28]. We discovered that BiP-positive regions displayed significantly higher SEC23A staining level, suggesting that SEC23A was associated with ER stress (Fig. 2B and C). To determine the relationship between SEC23A and ER stress, we first tested whether ER stress influences SEC23A expression in GC cells. MKN45 and HGC27 cells were treated with the ER stress inducers dithiothreitol (DTT) and tunicamycin (TM). qRT-PCR and western blotting results revealed that both DTT and TM treatments induced significant increases in SEC23A expression (Fig. 2D-G; Fig. S1A and B). Moreover, treatment with 4-PBA, an ER stress inhibitor, apparently reduced SEC23A expression (Fig. 2H and I). Comparable outcomes were obtained when we used different ER stress inducers, such as 5-FU and H_2_O_2_, to mimic the ER stressors that GC cells typically encounter (Fig. 2J-L; Fig. S1C and D). Then we further conducted immunofluorescence staining of SEC23A in human normal stomach tissues with BiP, found the same spatial expression association between SEC23A and BiP (Fig. S1E and F). In the BiP-positive region, SEC23A and BiP fluorescence intensity showed a significant positive correlation (Fig S1G). Moreover, the normal stomach tissues displayed lower expression of SEC23A and BiP than GC tissues (Fig. S1H and I). There was a positive correlation between SEC23A and BiP expression (Fig. S1J). Based on the results, we concluded that SEC23A was upregulated in response to ER stress and the increased ER stress may contributed to the high expression of SEC23A in GC tissues. Then we constructed stable SEC23A overexpressing or SEC23A knockdown GC cells using lentiviruses to explore whether SEC23A regulates ER stress (Fig. S1K and L). According to western blotting data, SEC23A knockdown increased BiP expression (Fig. S1M), whereas SEC23A overexpression decreased BiP expression, indicating that SEC23A may reduce the severity of ER stress (Fig. S1N). The expression relationship between SEC23A and BiP demonstrated by western blotting contradicts the results described above. We hypothesized that ER stress increases SEC23A expression, which serves in turn as a protective mechanism to relieve ER stress and promotes GC cells ER stress resistance.

### ER stress activated SEC23A transcription via the JAK2-STAT3 signaling pathway

**Fig. 3:**
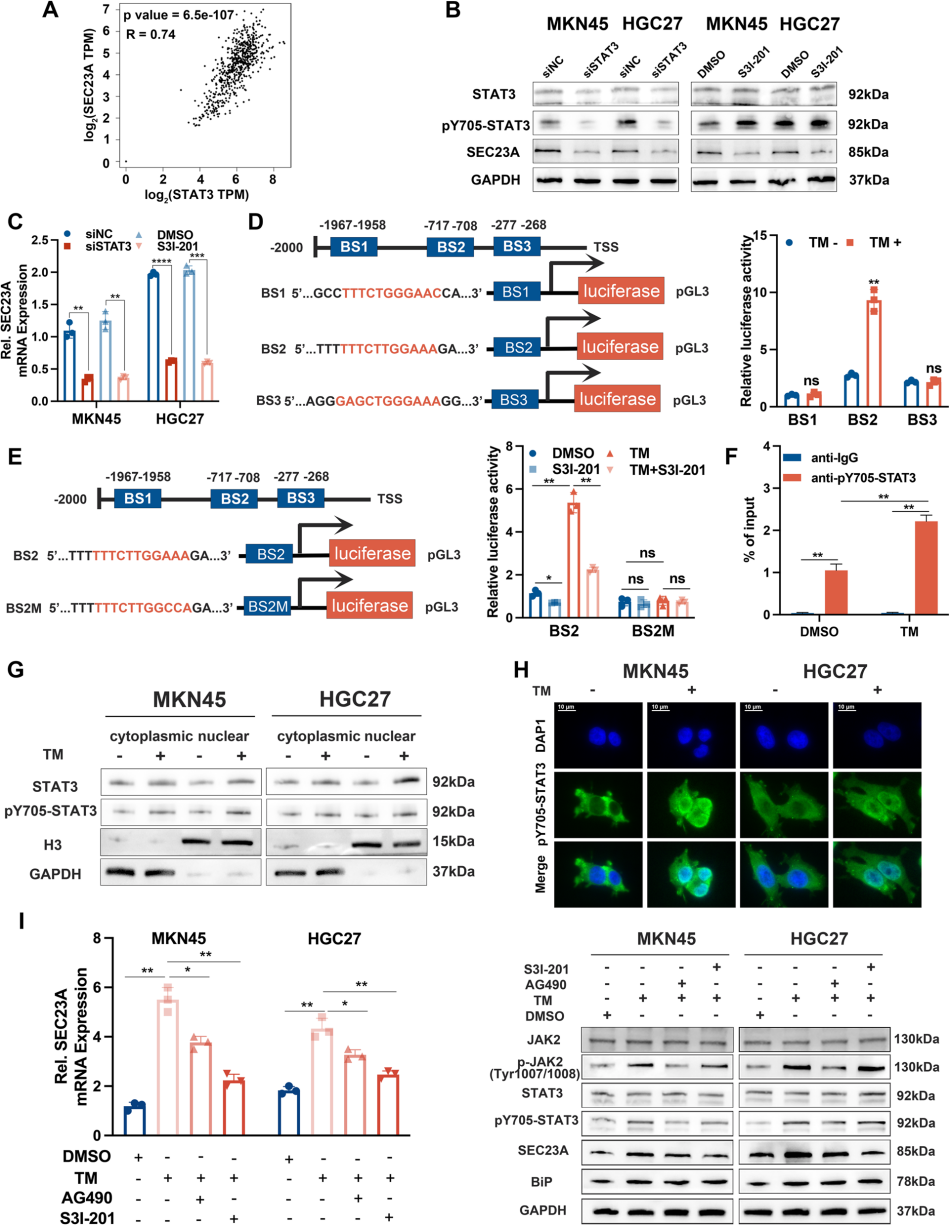
ER stress promoted SEC23A transcription by activating JAK2-STAT3 signaling pathway. **A** Spearman correlation analysis between STAT3 and SEC23A mRNA expression using TCGA dataset. **B **and** C** Western blotting (**B**) and qRT-PCR (**C**) assays to detect SEC23A expression in TM (1.0 µg/ml, 12 h) treated MKN45 and HGC27 cells with STAT3 knockdown or inhibited (S3I-201, 10 µM, 24 h) and corresponding controls. **D** Potential three STAT3 motif sites occupied in the promoter of SEC23A were cloned into luciferase reporter vectors (including BS1, BS2 and BS3) (left). Promoter-luciferase reporter activity changes of the three vectors in MKN45 cells after TM treatments (1.0 µg/ml, 12 h) (right). **E** The wild-type (BS2) and mutant form (BS2M) sequences of luciferase reporter vector containing the second binding site (left). Promoter luciferase reporter activity in MKN45 cells treated with indicated experimental settings (right). For TM treatments, 1.0 µg/ml, 12 h. For S3I-201 treatments, 10 µM, 24 h. **F** ChIP-qPCR analysis using anti-pY705-STAT3 antibody to determine the occupation of pY705-STAT3 on the SEC23A promoter in MKN45 cells in the presence or absence of 1.0 µg/ml TM treatment for 12 h. **G** Western blotting analysis to detect the subcellular expression of STAT3 and pY705-STAT3 in MKN45 and HGC27 cells with or without 1.0 µg/ml TM treatment for 12 h. **H** Immunofluorescence to detect the expression and localization of pY705-STAT3 in MKN45 and HGC27 cells with or without 1.0 µg/ml TM treatment for 12 h. **I** qRT-PCR and Western blotting assays to analysis the effect of JAK2 specific inhibitor AG490 and STAT3 inhibitor S3I-201 on the expression of SEC23A and JAK2/STAT3 signaling molecules in MKN45 and HGC27 cells under TM treatment (1.0 µg/ml, 12 h)

Previous studies have shown that cells activate the UPR in response to ER stress, including IRE1a, ATF6a and PERK pathways [29]. Due to the crosstalk between these UPR pathways, it is inappropriate to investigate the mechanism by which ER stress promotes SEC23A expression by only blocking only one of them. TM treatments increased SEC23A expression at both mRNA and protein levels, indicating that ER stress may stimulate SEC23A transcription. We first verified whether the several critical transcription factors in the UPR were upstream of SEC23A, including ATF4, ATF6, XBP1, CHOP, and CREB3L2. The findings demonstrated that suppression of these transcription factors had no discernible effect on SEC23A expression during ER stress (Fig. S2A-E). Then we explored the transcription factors that could potentially associate with the promoter region of SEC23A using the UCSC genome browser and JASPAR database. We found STAT3 may bind to the SEC23A promoter, and there was a significantly positive correlation between STAT3 and SEC23A mRNA expression in GC using GEPIA2 database (Fig. 3A). ER stress has been reported to be associated with the nuclear accumulation of pY705-STAT3 via the phosphorylation of PERK and subsequent activation of the JAK2-STAT3 pathway [30, 31]. To verify the involvement of this pathway in ER stress-induced SEC23A expression, we inhibited STAT3 using siRNA in GC cells and detected SEC23A expression with TM treatment. As expected, suppression of STAT3 expression attenuated ER stress-induced SEC23A upregulation, STAT3 inhibitor S3I-201 treatment produced the same outcomes (Fig. 3B, C). According to JASPAR database predicted results, three classical STAT3 binding motifs were identified in the promoter of SEC23A (2000 bp upstream of the transcription start site). Therefore, we constructed three luciferase reporter plasmids named BS1, BS2 and BS3, containing these motifs to further determine whether STAT3 transcriptionally regulates SEC23A (Fig. 3D). Only BS2 exhibited increased luciferase activity upon TM treatment, and this increase was diminished by STAT3 inhibition (Fig. 3D and E). The regulation of BS2 luciferase activity following TM treatment was blocked in the mutant plasmid, indicating that the second binding site was a positive STAT3 binding site on the SEC23A promoter (Fig. 3E). ChIP-qPCR assays showed that STAT3 could directly bind to the SEC23A promoter region, and the occupation of STAT3 on the SEC23A promoter was increased following TM treatment (Fig. 3F). Taken together, our data indicated that STAT3 was an upstream transcriptional factor of SEC23A during ER stress. Consistent with reported results, TM treatment promoted STAT3 phosphorylation at Tyr705 in both the cytoplasm and the nucleus, as shown by western blotting (Fig. 3G) and immunofluorescence confocal microscopy (Fig. 3H). Furthermore, increased SEC23A expression resulting from ER stress was diminished by JAK2 inhibitor AG490 and STAT3 inhibitor S3I-201 treatments (Fig. 3I). Taken together, these data indicated that activation of the JAK2-STAT3 pathway contributes to ER stress-induced SEC23A upregulation in GC cells.

### SEC23A promoted GC cell resistance to ER stress

**Fig. 4:**
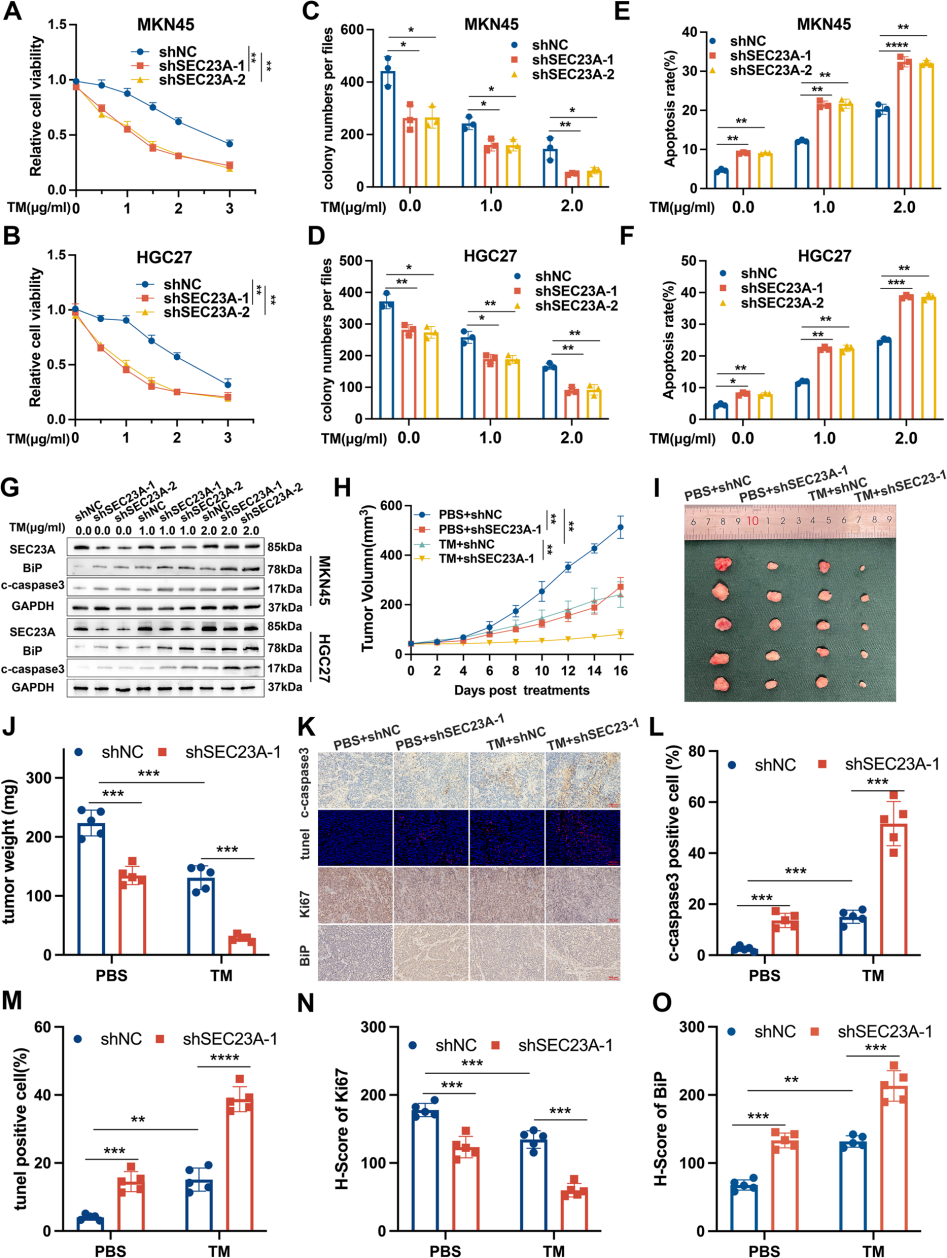
SEC23A promoted GC cells resistance to ER stress in vitro and vivo. **A **and** B** CCK 8 assays in SEC23A silenced MKN45 cells (**A**) and HGC27 cells (**B**) under concentration gradient TM treatments for 12 h. **C **and** D** Quantification of colony formation assays to determine cell survival in SEC23A silenced MKN45 (**C**) and HGC27 cells. **D** treated with indicated doses of TM for 12 h. **E **and** F** Quantification of flow cytometry to analysis cell apoptosis in SEC23A silenced MKN45 (**E**) and HGC27 cells (**F**) treated with indicated doses of TM for 12 h. **G** Western blotting against c-caspase3 performed on SEC23A silenced MKN45 and HGC27 cells under 12 h TM treatment with indicated doses. **H** Changes in subcutaneous tumor volumes of mice from the four groups (including PBS + shNC, PBS + shSEC23A-1, TM + shNC, TM + shSEC23A-1 groups). **I **and** J** Quantification of subcutaneous tumor weight (**I**) and the tumor representative images (**J**) from the four groups. **K** Subcutaneous tumor section images from the four groups stained with anti-c-caspase3 antibody, tunel reagent, anti-Ki67 antibody and BiP antibody. **L-O** Quantification of c-caspase3 (**L**), Tunel (**M**), Ki67 (**N**) and BiP (**O**) stain of the four groups

We conducted additional functional experiments to ascertain whether increased SEC23A expression is a factor that protects GC cells from ER stress-induced apoptosis. As determined by CCK-8 and colony formation assays, SEC23A suppression significantly decreased cell viability in GC cells treated with TM (Fig. 4A-D), whereas SEC23A overexpression significantly increased GC cell resistance to ER stress (Fig. S3A-C). The effect of SEC23A on the apoptosis of TM-treated cells was further verified by flow cytometry using annexin V-PI labeling and western blotting against c-caspase3 and c-PARP (Fig. 4E-G; Fig. S3D-H). Then we subcutaneously inoculated the shNC and shSEC23A-1 MKN45 cells into nude mice to verify the roles of SEC23A in GC growth and ER stress adaptation in vivo. Four experimental groups were established: PBS + shNC, PBS + shSEC23A-1, TM + shNC, and TM + shSEC23A-1. The tumor volume and weight showed that SEC23A knockdown could inhibit tumor growth and enhance MKN45 cells sensitivity to ER stress in vivo (Fig. 4H-J). C-caspase3 and BiP staining and Tunel assays showed that the shSEC23A-1 group with or without TM treatments exhibited more apoptotic cells and ER stress severity, suggesting that shSEC23A-1 cells were more susceptible to apoptosis and more sensitive to ER stress than shNC cells in vivo (Fig. 4K-M and O). The immunohistochemistry results of the shSEC23A-1 group revealed less Ki-67 staining in tumor tissues than the shNC group, with or without TM treatment (Fig. 4K and N). In conclusion, SEC23A promoted GC cells’ survival advantage during ER stress, whether from baseline or extra stimuli.

### SEC23A contributed to the autophagy activation in GC cells during ER stress

**Fig. 5:**
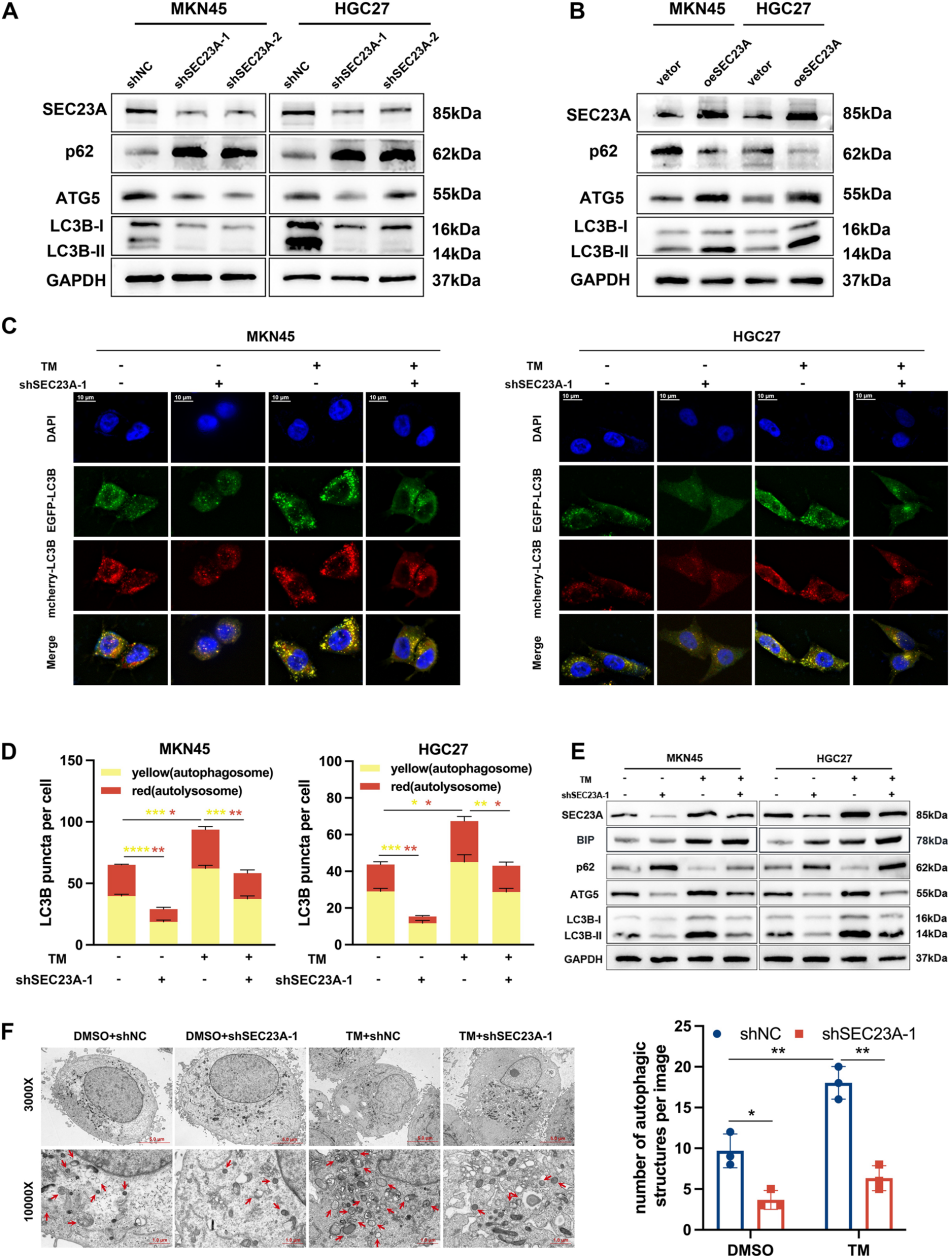
SEC23A contributed to the activated autophagy in GC cells during ER stress. **A** Expression levels of p62, ATG5, LC3B-I and LC3B-II in MKN45 and HGC27 cells with SEC23A knockdown and corresponding controls detected by western blotting. **B** Expression levels of p62, ATG5, LC3B-I and LC3B-II in MKN45 and HGC27 cells with SEC23A overexpression and corresponding control detected by western blotting. **C **and** D** Representative immunofluorescence images (**C**) and quantification (**D**) of the LC3B puncta in mCherry-EGFP-LC3B transfected MKN45 and HGC27 cells with SEC23A knockdown and corresponding controls in the presence or absence of 1.0 µg/ml TM treatment for 12 h. **E** Western blotting results of p62, ATG5, LC3B-I and LC3B-II expression levels in MKN45 and HGC27 cells with SEC23A knockdown and corresponding controls in the presence or absence of 1.0 µg/ml TM treatment for 12 h. **F** Representative autophagic structure images (left) and quantification (right) of transmission electron microscopy in shNC and shSEC23A-1 MKN45 cells with or without 1.0 µg/ml TM treatment for 12 h

Previous studies have reported that SEC23A is involved in elevating autophagy [26]. Autophagy has been linked to promotion of ER stress adaptation in cancer cells [32, 33]. Therefore, we explored whether SEC23A enhanced autophagy to protect GC cells from ER stress-induced cell death. Western blotting revealed that the shSEC23A-1 and shSEC23A-2 cells displayed a lower expression of LC3B-II and ATG5, and a higher expression of SQSTM2/p62 than the shNC cells (Fig. 5A). Consistently, the oeSEC23A GC cells exhibited higher LC3B-II and ATG5, lower p62 expression compared to the vector GC cells (Fig. 5B). Confocal microscopy observations demonstrated that SEC23A knockdown decreased autophagosomes and autolysosomes numbers (Fig. 5C and D). SEC23A overexpression elevated the accumulation of autophagosomes and autolysosomes (Fig. S4A and B). These results suggested that SEC23A enhanced basal autophagic flux in GC cells. Furthermore, TM treatments induced significant increases in the number of autophagosomes and autolysosomes, whereas SEC23A suppression attenuated the accumulation of autophagosomes and autolysosomes induced by TM treatment (Fig. 5C and D). Moreover, western blotting results showed the increased LC3B-II and ATG5, decreased p62 following TM treatments were reversed by SEC23A suppression (Fig. 5E). The same results were obtained through electron microscopy observation (Fig. 5F). Accordingly, it was concluded that SEC23A contributed to the autophagy activation in GC cells under ER stress.

### SEC23A increased ANXA2 localization on the cell membrane to stimulate autophagy

**Fig. 6:**
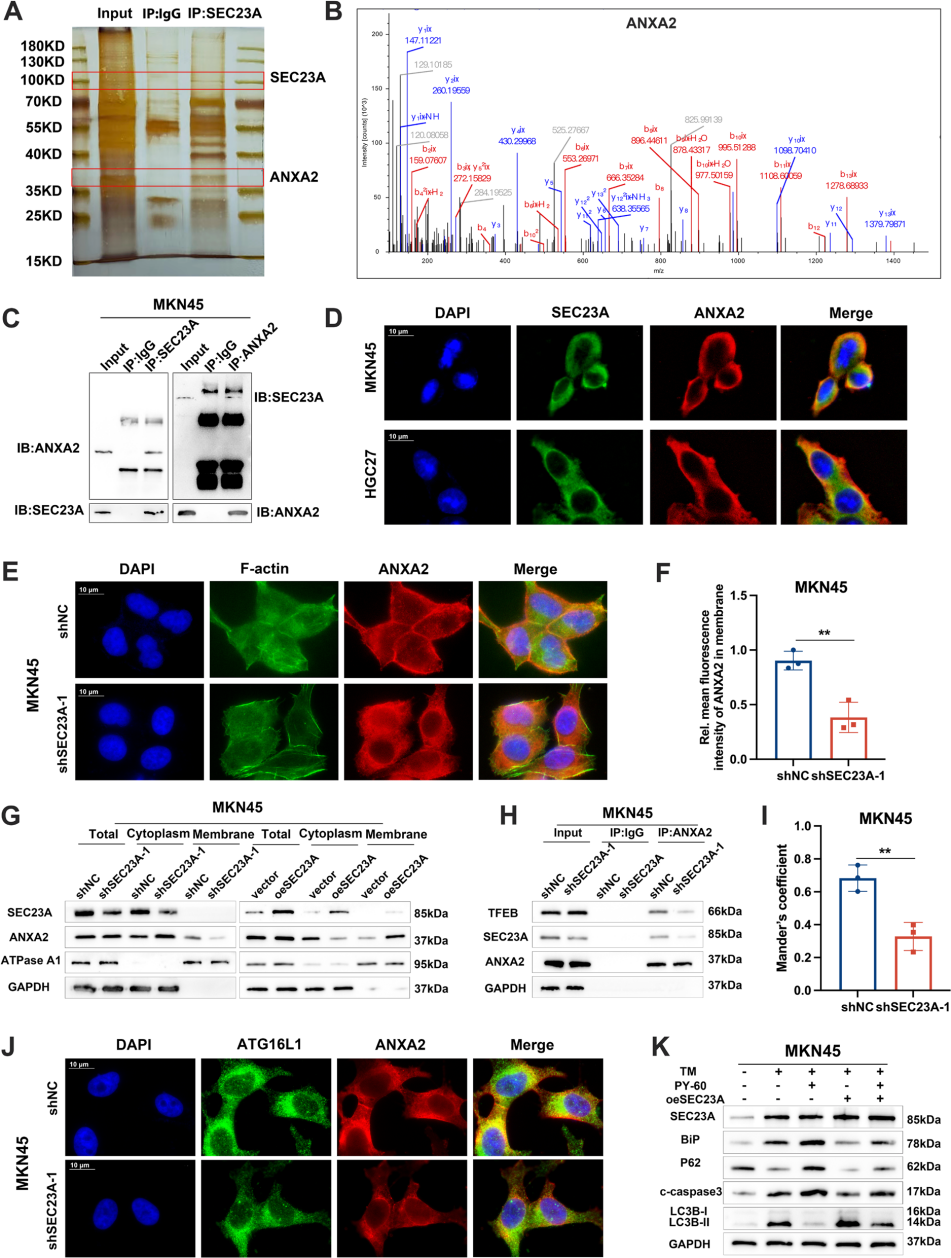
SEC23A increased ANXA2 localization on the cell membrane to stimulate autophagy. **A** Silver staining image of IP assay using anti-SEC23A antibody and IgG in MKN45 cells. **B** Mass spectrometry image of ANXA2. **C** Co-IP assay using anti-SEC23A and anti-ANXA2 antibody to detect the binding of SEC23A and ANXA2 in MKN45 cells. **D** Immunofluorescence co-staining SEC23A and ANXA2 in MKN45 and HGC27 cells. **E** and **F** Immunofluorescence images (**E**) and quantitation (**F**) to detect the cell membrane localization of ANXA2 in shNC and shSEC23A-1 MKN45 cells. **G** Western blotting analysis to detect the subcellular expression of ANXA2 in shNC and shSEC23A-1 MKN45 cells. **H** IP assay using anti-ANXA2 antibody to detect the content of TFEB bound to ANXA2 in shNC and shSEC23A-1 MKN45 cells. **I** and **J** Immunofluorescence quantitation (**I**) and images (**J**) to detect the co-localization of ANXA2 and ATG16L1 in shNC and shSEC23A-1 MKN45 cells. **K** Western blotting analysis to detect the expression of LC3B-I, LC3B-II, p62, BiP, c-caspase3 in MKN45 cell with indicated experimental settings

To explore the mechanisms by which SEC23A induces autophagy in GC cells undergoing ER stress, we preformed IP assay and mass spectrometry to identify the proteins which SEC23A interacts in MKN45 cells under ER stress (Fig. 6A). According to the results, Annexin A2 (ANXA2) was bound to SEC23A with the highest score (Fig. 6B). Co-IP and immunofluorescence assays further validated the binding and colocalization of SEC23A and ANXA2 (Fig. 6C and D; Fig. S5A). ANXA2 is an annexin family protein involved in multiple biological processes, including epithelial–mesenchymal transition, chemoresistance and autophagy [34–36]. We hypothesized that ANXA2 contributes to SEC23A induced autophagy and ER stress relief. Western blotting results showed that knockdown or overexpression of SEC23A had no effect on ANXA2 expression during ER stress (Fig. S5B). Previous research has demonstrated that the spatial localization of ANXA2 affects how it functions. Cytoplasmic ANXA2 prevents autophagy by combining with TFEB, a dominant transcription factor for autophagy, and rendering it inactive [34]. While ANXA2, which is located at the plasma membrane, promotes autophagy by boosting the production of plasma membrane-derived ATG16L^+^ autophagosomes [35]. We speculated that SEC23A regulated the subcellular localization of ANXA2. Subcellular fractionation western blotting and cellular immunofluorescence performed on TM-treated GC cells, we first demonstrated that ANXA2 was predominantly localized in the cytoplasm when SEC23A was suppressed, whereas ANXA2 cell membrane localization was dramatically increased when SEC23A was overexpressed, indicating that SEC23A facilitated localization of ANXA2 on cell membranes during ER stress (Fig. 6E-G; Fig. S5C-E). The interaction between ANXA2 and TFEB was decreased following SEC23A knockdown (Fig. 6H; Fig. S5F). SEC23A knockdown reduced the colocalization between ANXA2 and ATG16L1, indicating SEC23A knockdown may inhibit the production of ATG16L^+^ autophagosomes by regulating ANXA2 plasma membrane trafficking (Fig. 6I and J; Fig. S5G and H). Then we treated oeSEC23A GC cells with PY-60, a chemical that liberates ANXA2 from the plasma membrane by inhibiting its binding phosphoinositide [37]. The protection during ER stress induced by SEC23A was attenuated by PY-60 treatment (Fig. S5I). PY-60 treatment decreased LC3B-II and increased p62 expression in TM treated GC cells, and PY-60 reversed the SEC23A-induced decreases in BiP, c-caspase3 and c-PARP expression (Fig. 6K; Fig. S5J and K). Collectively, these findings suggested that SEC23A promotes autophagy and ER stress relief by regulating the localization of ANXA2 at the plasma membrane.

### SEC23A protected GC cells from ER stress-induced apoptosis by promoting autophagy

**Fig. 7:**
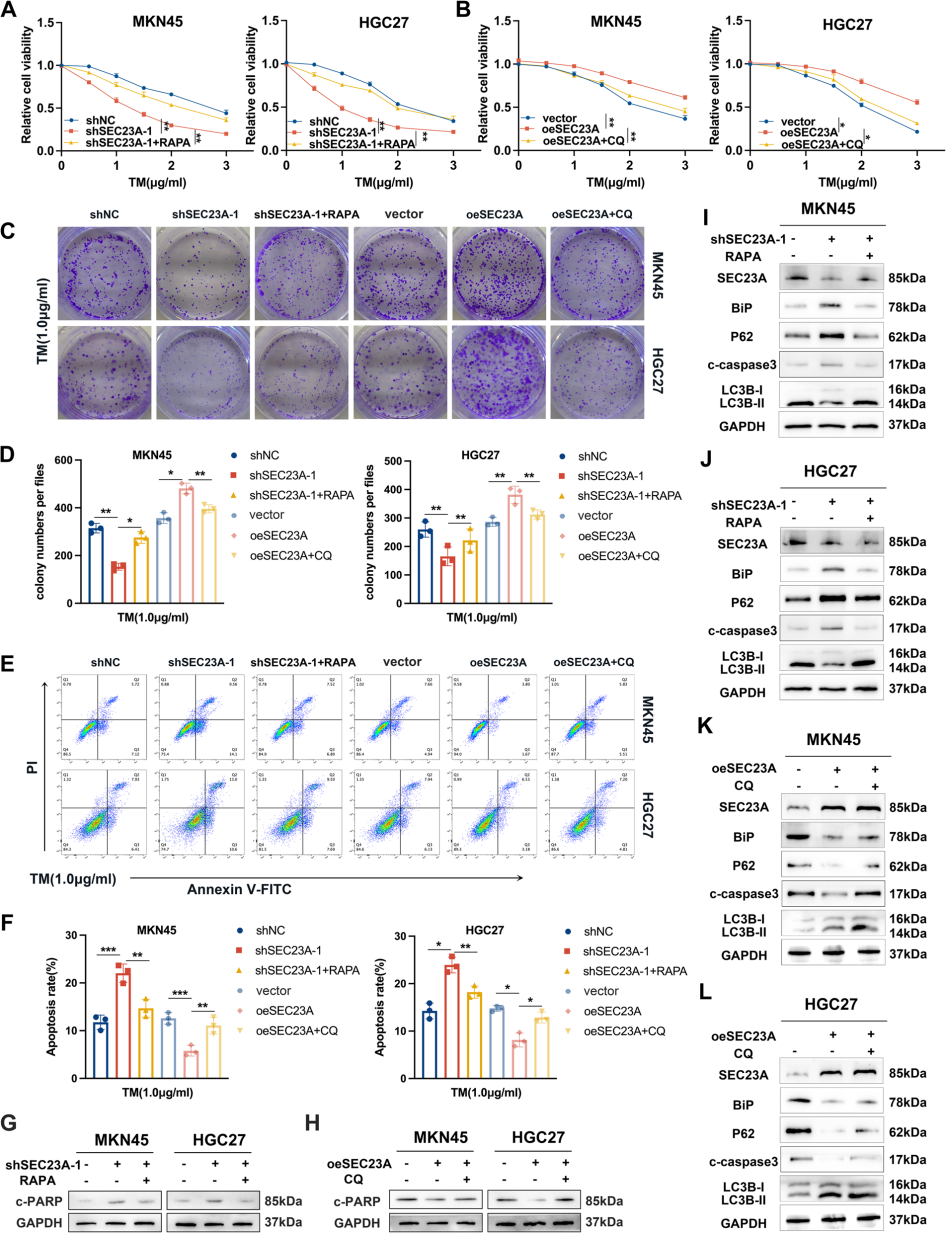
Autophagy was contributed to ER stress adaptation induced by SEC23A in GC cells. **A** CCK8 assays of three groups (including shNC, shSEC23A-1 and shSEC23A-1 + RAPA [15 µM, 12 h]) in MKN45 cells and HGC27 cells under concentration gradient TM treatments for 12 h. **B** CCK8 assays of three groups (including vector, oeSEC23A and oeSEC23A + CQ [10 µM, 12 h]) in MKN45 cells and HGC27 cells under concentration gradient TM treatments for 12 h. **C **and** D** Colony formation assays images (**C**) and quantification (**D**) of six groups (including shNC, shSEC23A-1, shSEC23A-1 + RAPA [15 µM, 12 h], vetor, oeSEC23A and oeSEC23A + CQ [10 µM, 12 h]) in MKN45 and HGC27 cells under indicated TM treatments for 12 h. **E **and** F** Flow cytometry images (**E**) and quantification (**F**) of the six groups in MKN45 cells and HGC27 cells under indicated TM treatments for 12 h. **G **and** H** Western blotting against c-PARP to analysis the effects of RAPA (**G**) and CQ (**H**) on apoptosis in MKN45 cells and HGC27 cells with SEC23A knockdown or overexpression under 1.0 µg/ml TM treatments for 12 h. **I-L** Western blotting against c-caspase3 and BiP to analysis the effects of RAPA (**I **and** J**) and CQ (**K **and** L**) on apoptosis in MKN45 cells and HGC27 cells with SEC23A knockdown or overexpression under 1.0 µg/ml TM treatments for 12 h

To confirm the contribution of autophagy to ER stress survival advantage conferred by SEC23A, we severally applied the autophagy activator RAPA on shSEC23A GC cells and the autophagy inhibitor CQ on oeSEC23A GC cells. Results from the CCK8 assay revealed that shSEC23A-1 + RAPA group performed with greater cell viability than the shSEC23A group following TM treatment (Fig. 7A). After receiving CQ treatment, the cell viability of oeSEC23A group was dramatically decreased (Fig. 7B). Colony formation assays produced comparable findings (Fig. 7C and D). We next conducted flow-cytometric apoptosis assays and western blotting against c-caspase3 and c-PARP to detect cell apoptosis. RAPA treatments reversed the increased cell apoptosis observed in shSEC23A GC cells (Fig. 7E-G, I and J). Moreover, CQ treatments decreased ER stress resistance in oeSEC23A GC cells (Fig. 7E, F, H, K and L). These results further verified that autophagy contributes to ER stress adaptation induced by SEC23A in GC cells.

### SEC23A reduced chemotherapeutic efficacy via autophagy-mediated ER stress adaptation

**Fig. 8:**
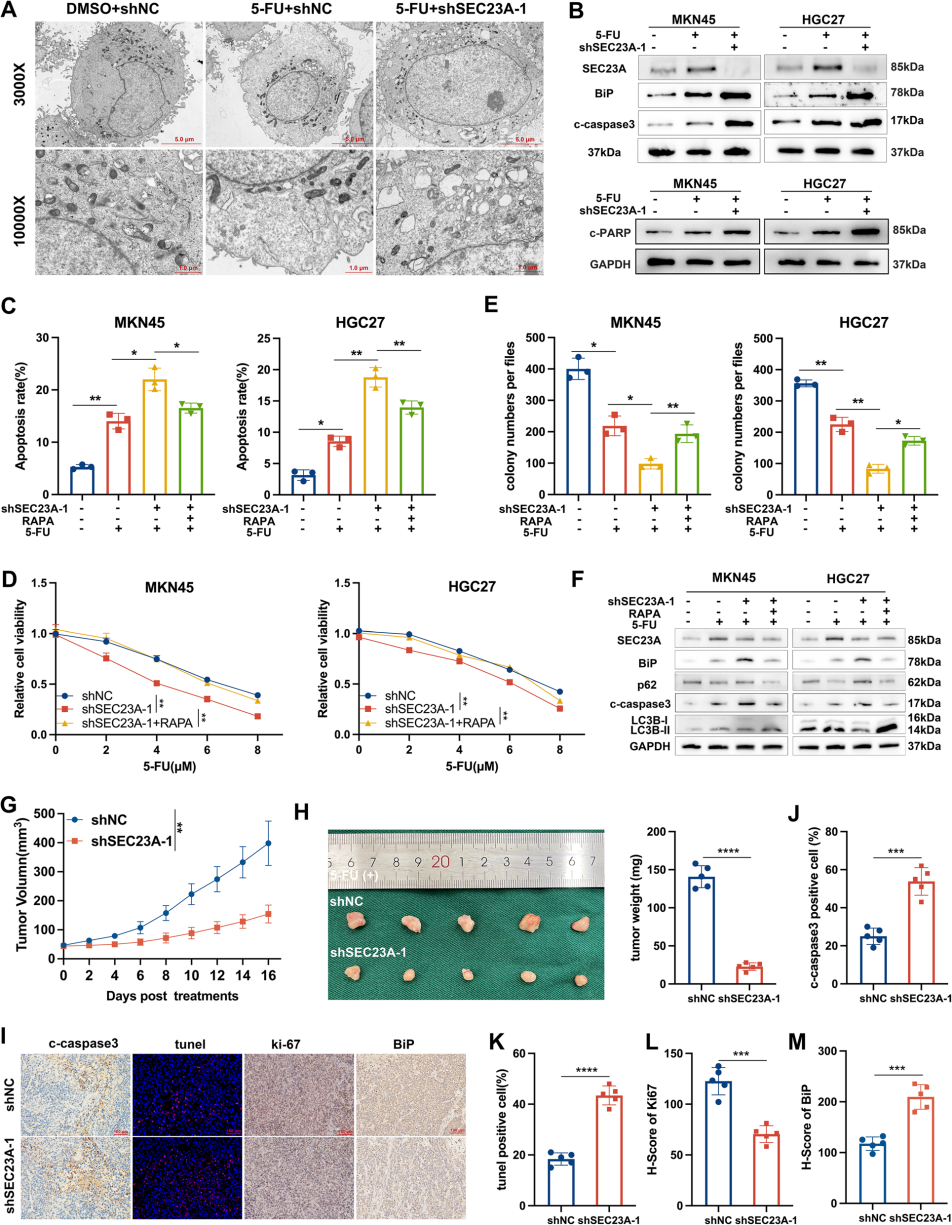
SEC23A protected gastric cancer cells against 5-FU induced cell death through enhancing autophagy. **A** Representative endolpasmic reticulum images of transmission electron microscopy in indicated MKN45 cells. **B** Western blotting to detect c-caspase3 (upper) and c-PARP (lower) expression in indicated GC cells. **C** Flow cytometry quantification of the indicated groups in MKN45 and HGC27 cells with or without 4 µM 5-FU treatments for 12 h. **D** CCK8 assays of three groups (including shNC, shSEC23A-1 and shSEC23A-1 + RAPA [15 µM, 12 h]) in MKN45 cells (left) and HGC27 cells (right) under concentration gradient 5-FU for 12 h. **E** Colony formation assays quantification of the indicated groups in MKN45 and HGC27 cells with or without 4 µM 5-FU treatments for 12 h. **F** Western blotting against c-caspase3 and BiP in the indicated groups in MKN45 and HGC27 cells with or without 4 µM 5-FU treatments for 12 h. **G** Subcutaneous tumor volumes in mice injected with shNC or shSEC23A-1 MKN45 cells under 5-FU treatment. (**H**) Tumor representative images (left) and quantification of subcutaneous tumor weight (right) from indicated groups. **I-M** Subcutaneous tumor section images (**I**) and quantification from the two groups stained with anti-c-caspase-3 antibody (**J**), tunel reagents (**K**), anti-Ki67 antibody (**L**), anti-c-PARP antibody (**M**)

Anticancer drugs, such as sorafenib, bortezomib, cisplatin, 5-FU, and paclitaxel have been shown to cause cancer cell death by triggering ER stress-mediated apoptosis [38–42]. Various tumors acquire chemoresistance as a result of ER stress adaptation [9, 43, 44]. Resistance to chemotherapy can be lessened by treatments that target ER stress [45–50]. Inhibition of cytoprotective autophagy could enhance chemotherapeutic efficacy [51–54]. Considering the roles that SEC23A played in ER stress relief and autophagy, we next investigated whether SEC23A influences the effectiveness of chemotherapy. We treated GC cells with 5-FU, a first-line chemotherapeutic agent against GC [1]. Cell transmission electron microscopy and western blotting experiments indicated that 5-FU triggered ER stress, and knockdown of SEC23A further increased the severity of ER stress induced by 5-FU (Fig. 8A and B). To unveil the possible link connecting SEC23A and chemotherapy effect, we conducted functional experiments in vitro and vivo. SEC23A knockdown significantly sensitized GC cells to 5-FU and overexpressed SEC23A decreased 5-FU-induced cell death, as evidenced by flow cytometry and cell viability (Fig. 8C and D; Fig. S6A-C). Compared to shSEC23A-1 group, autophagy activator RAPA reversed the decreased cell viability and increased cell apoptosis rate (Fig. 8C and D; Fig. S6A). Furthermore, autophagy inhibitor CQ apparently weakened cell viability and increased cell apoptotic rate in SEC23A overexpressed GC cells treated with 5-FU (Fig. S6A-C). The colony formation assays and western blotting against c-caspase3 and c-PARP obtained similar results (Fig. 8E and F; Fig. S6D-H). Under 5-FU treatments, SEC23A knockdown inhibited tumor growth, as demonstrated by the volume and weight of tumors (Fig. 8G and H). These results were also validated by Ki67, c-caspase3, BiP and tunel staining (Fig. 8I-L). Combined with the previous findings, we concluded that SEC23A attenuated 5-FU efficacy through autophagy-induced ER stress relief in GC cells.

## Discussion

ER stress is implicated in multiple biological processes during GC tumorigenesis and progression, including cell proliferation, metastasis, and therapy resistance [6]. Moderate ER stress promotes the malignant biological behaviors of GC cells, but when the severity of ER stress and the cell protective mechanisms are out of balance, apoptosis will occur [5]. It is essential to investigate the mechanisms by which GC cells resist apoptosis under conditions of ER stress in order to prevent tumor progression and enhance therapeutic efficacy. Autophagy has been reported to be involved in ER stress adaptation and therapeutic resistance [13, 16]. Our study found that during ER stress, pY705-STAT3 activated SEC23A transcription and SEC23A protected GC cells from ER stress-induced apoptosis by promoting autophagy. The ER stress-SEC23A-autophagy negative feedback loop may help gastric cancer adapt to the unfavorable survival environments during its development by alleviating ER stress, which represent a potential target for inhibiting GC progression.

In this study, we revealed a novel function and underlying mechanisms of SEC23A in GC. SEC23A has been reported to be involved in several cancers [26, 55]. Recent studies demonstrated that SEC23A was associated with immune infiltration and poor prognosis in GC [24, 56]. According to our results, SEC23A was highly expressed in GC tissues and predicted a poor prognosis in GC patients. ER stress induced pY705-STAT3 contributed to the upregulation of SEC23A in GC. Whether other factors are involved in the increased expression of SEC23A in GC requires further investigation. Highly expressed SEC23A increased autophagy by promoting the plasma membrane localization of ANXA2. The SEC23A-ANXA2-autophagy axis, in turn, relieved ER stress and decreased the sensitivity of GC cells to ER stress. Disruption of the SEC23A-ANXA2-autophagy axis may suppress GC progression by decreasing the resistance to ER stress. Moreover, we revealed a novel mechanism by which SEC23A facilitates autophagy. Previous studies have demonstrated that SEC23A increases autophagy by promoting S100A8 autocrine in melanoma [26]. We found that SEC23A interacted with ANXA2 and promoted its translocation to plasma membrane. Consistent with previous studies [34, 35], we verified the membrane-localized ANXA2 resulting from SEC23A promoted autophagy by increasing the formation of ATG16L^+^ autophagosomes and decreasing its combination with TFEB. But how SEC23A regulates the cellular localization of ANXA2 requires additional investigation.

In addition, we further investigated that SEC23A negatively regulated 5-FU efficacy by autophagy-mediated ER stress relief. Various tumors acquire therapy resistance as a result of ER stress adaptation [43, 44]. Recent studies reported that ID1 conferred ovarian cancer cell chemoresistance through ER stress-mediated induction of autophagy [57], and RSK2 suppression increased paclitaxel induced apoptosis by regulating autophagy [33]. Considering that 5-FU-based regimens are the first line chemotherapy treatments against GC and 5-FU is associated with ER stress and autophagy [27, 50], we explored the influence of SEC23A on 5-FU efficacy in GC cells. We found that 5-FU triggers ER stress and promoted SEC23A expression. SEC23A suppression exacerbated 5-FU induced ER stress and cell death by regulating autophagy. These results indicated that ER stress-SEC23A-autophagy axis suppression could provide an opportunity to enhance chemotherapy effectiveness in GC patients with chemoresistance. However, the current research focused solely on the effect of SEC23A on the 5-FU efficacy. The relationship between SEC23A and other treatment efficacies for GC requires further study.

## Conclusions

In summary we revealed a negative feedback loop consisting of ER stress, SEC23A and autophagy. ER stress promotes SEC23A transcriptional upregulation and the subsequent SEC23A-ANXA2-autophagy signaling axis, in turn, relieves ER stress and maintains survival advantage in GC cells during ER stress. Consequently, based on our findings, SEC23A may represent a promising molecular target for prognostic prediction and potential therapeutic intervention in patients with GC.

### Web links and URLs

GEPIA2: http://gepia2.cancer-pku.cn/

JASPAR database: https://jaspar.genereg.net/

UCSC genome browser: http://genome.ucsc.edu/

### Supplementary Information

**Additional file 1:Table S1. **Sequences used for RNAi. **Table S2.** Primers used for qRT-PCR. **Table S3.** Antibodies used for western blotting. **Table S4.** Reagents used in the experiments. 

**Additional file 2:** **Figure S1.** Additional results of Figure 2. (**A**) qRT-PCR (left) and western blotting (right) analysis of SEC23A expression in HGC27 cells under concentration gradient treatments of DTT for 12 h. (**B**) qRT-PCR (left) and western blotting (right) analysis of SEC23A expression in HGC27 cells under concentration gradient treatments of TM for 12 h. (**C**) qRT-PCR (upper) and western blotting (lower) analysis of SEC23A expression in HGC27 cells under concentration gradient treatments of H_2_O_2_ for 24 h. (**D**) qRT-PCR (upper) and western blotting (lower) analysis of SEC23A expression in HGC27 cells under concentration gradient treatments of 5-FU for 24 h. (**E**) Representative images of immunofluorescence co-staining SEC23A, BiP and cytokeratin in 15 GC paratumor tissues. (**F**) Fluorescence intensity quantitative analysis of SEC23A expression in BiP^+^ and BiP^-^ areas of human GC paratumor tissues (*n* = 15). (**G**) Correlation between SEC23A and BiP fluorescent intensity in BiP^+^ areas of 15 paired GC and adjacent normal tissues. (*n*= 30). (H) Representative images of SEC23A and BiP western blotting results in 30 paired GC and adjacent normal tissues. (**I**) Quantitative analysis of SEC23A and BiP western blotting results in 30 paired GC and adjacent normal tissues. (**J**) Correlation between SEC23A and BiP protein expression in 30 paired GC and adjacent normal tissues. (*n* = 60). (**K** and **L**) Knockdown and overexpression efficiencies were verified in MKN45 and HGC27 cells by qRT-PCR (K) and western blotting (**L**) assays. (**M** and **N**) Western blotting assays to analysis the expression of BiP in the condition of SEC23A knockdown (**M**) or overexpression (**N**). **Figure S2.** Additional results of Figure. (**A-E**) Western blotting to analysis SEC23A expression changes in MKN45 and HGC27 cells under TM treatment (1.0 µg/ml, 12 h) after knockdown ATF4 (**A**), ATF6 (**B**), XBP1 (**C**), CHOP (**D**) and CREB3L2 (**E**) respectively. **Figure S3.** Additional results of Figure 4. (**A**) CCK 8 assays in MKN45 cells and HGC27 cells when SEC23A was overexpressed under concentration gradient TM treatments for 12 h. (**B**) Quantification of colony formation assays to determine cell survival in SEC23A overexpressed MKN45 and HGC27 cells treated with indicated doses of TM for 12 h. (**C **and** D**) Representative images of colony formation assays and flow cytometry in TM (indicated doses, 12 h) treated MKN45 and HGC27 cells with SEC23A knockdown, overexpression and corresponding control. (**E**) Quantification of flow cytometry to analysis cell apoptosis in SEC23A overexpressed MKN45 and HGC27 cells treated with indicated doses of TM for 12 h. (**F**) Western blotting against c-PARP performed on SEC23A silenced MKN45 and HGC27 cells under 12 h TM treatment with indicated doses. (**G **and** H**) Western blotting against c-caspase3 (**G**) and c-PARP (**H**) performed on SEC23A overexpressed MKN45 and HGC27 cells under 12 h TM treatment with indicated doses. **Figure S4.** Additional results of Figure 5. (**A**) Representative immunofluorescence images of the mCherry-EGFP-LC3B transfected MKN45 and HGC27 cells with SEC23A overexpression and corresponding controls. (**B**) Quantification of the mCherry-EGFP-LC3B transfected MKN45 and HGC27 cells with SEC23A overexpression and corresponding controls. **Figure S5.** Additional results of Figure 6. (**A**) Co-IP assay using anti-SEC23A and anti-ANXA2 antibody to detect the binding of SEC23A and ANXA2 in HGC27 cells. (**B**) Western blotting assay to detect the expression of ANXA2 in MAN45 and HGC27 cells in the condition of SEC23A knockdown or expression. (**C **and** D**) Immunofluorescence images (**C**) and quantitation (**D**) to detect the cell membrane localization of ANXA2 in shNC and shSEC23A-1 HGC27 cells. (**E**) Western blotting analysis to detect the subcellular expression of ANXA2 in shNC and shSEC23A-1 HGC27 cells. (**F**) IP assay using anti-ANXA2 antibody to detect the content of TFEB bound to ANXA2 in shNC and shSEC23A-1 HGC27 cells. (**G **and** H**) Immunofluorescence quantitation (**G**) and images (**H**) to detect the co-localization of ANXA2 and ATG16L1 in shNC and shSEC23A-1 HGC27 cells. (**I**) CCK8 assays in MKN45 and HGC27 cells with indicated experimental settings. (**J**) Western blotting analysis to detect the expression of LC3B-I, LC3B-II, p62, BiP, c-caspase3 in HGC27 cells with indicated experimental settings. (**K**) Western blotting analysis to detect the expression of c-PARP in MKN45 and HGC27 cells with indicated experimental settings. **Figure S6.** Additional results of Figure 8. (**A**) Representative images of flow cytometry of the indicated groups. (**B**) CCK8 assays of three groups (including vetor, oeSEC23A and oeSEC23A+CQ [10 µM, 12 h] ) in MKN45 cells and HGC27 cells under concentration gradient 5-FU for 12 h. (**C**) Flow cytometry quantification of the indicated groups in MKN45 and HGC27 cells with or without 4 µM 5-FU treatments for 12 h. (**D**) Colony formation assays quantification of the indicated groups in MKN45 and HGC27 cells with or without 4 µM 5-FU treatments for 12 h. (**E**) Western blotting against c-caspase3 and BiP in the indicated groups in MKN45 and HGC27 cells with or without 4 µM 5-FU treatments for 12 h. (**F**) Representative images of Colony formation assays of the indicated groups. (**G**) Western blotting against c-PARP in the indicated groups in MKN45 and HGC27 cells with or without 4 µM 5-FU treatments for 12 h.

**Additional file 3.** Supplemental Methods.

**Additional file 4.** 

**Additional file 5.** 

## Data Availability

All the data used in the current study are available from the corresponding authors upon reasonable request.

## Abbreviation

5-FU: 5-Fluorocrail
ANXA2: Annexin A2
BiP: Binding-immunoglobulin protein
CQ: Chloroquine
ER: Endoplasmic reticulum
GC: Gastric cancer
IHC: Immunohistochemistry
RAPA: Rapamune
SEC23A: Sec23 homolog A
STAT3: Activator of transcription 3
TCGA: The Cancer Genome Atlas
TFEB: Transcription factor EB
TM: Tunicamycin
UPR: Unfolded protein response

## References

[1] Smyth EC, Nilsson M, Grabsch HI, van Grieken NC, Lordick F (2020). Gastric cancer. Lancet.

[2] Sung H, Ferlay J, Siegel RL, Laversanne M, Soerjomataram I, Jemal A (2021). Global cancer statistics 2020: GLOBOCAN estimates of incidence and mortality worldwide for 36 cancers in 185 Countries. CA Cancer J Clin.

[3] Shen L, Shan YS, Hu HM, Price TJ, Sirohi B, Yeh KH (2013). Management of gastric cancer in Asia: resource-stratified guidelines. Lancet Oncol.

[4] Cristescu R, Lee J, Nebozhyn M, Kim KM, Ting JC, Wong SS (2015). Molecular analysis of gastric cancer identifies subtypes associated with distinct clinical outcomes. Nat Med.

[5] Chen X, Cubillos-Ruiz JR (2021). Endoplasmic reticulum stress signals in the tumour and its microenvironment. Nat Rev Cancer.

[6] Wang M, Kaufman RJ (2014). The impact of the endoplasmic reticulum protein-folding environment on cancer development. Nat Rev Cancer.

[7] Walter P, Ron D (2011). The unfolded protein response: from stress pathway to homeostatic regulation. Science.

[8] Guo B, Xiong X, Hasani S, Wen YA, Li AT, Martinez R (2021). Downregulation of PHLPP induced by endoplasmic reticulum stress promotes eIF2alpha phosphorylation and chemoresistance in colon cancer. Cell Death Dis.

[9] Sniegocka M, Liccardo F, Fazi F, Masciarelli S (2022). Understanding ER homeostasis and the UPR to enhance treatment efficacy of acute myeloid leukemia. Drug Resist Updat.

[10] Zhu C, Xie Y, Li Q, Zhang Z, Chen J, Zhang K (2023). CPSF6-mediated XBP1 3’UTR shortening attenuates cisplatin-induced ER stress and elevates chemo-resistance in lung adenocarcinoma. Drug Resist Updat.

[11] Levy JMM, Towers CG, Thorburn A (2017). Targeting autophagy in cancer. Nat Rev Cancer.

[12] Yim WW, Mizushima N (2020). Lysosome biology in autophagy. Cell Discov.

[13] Dang TT, Kim MJ, Lee YY, Le HT, Kim KH, Nam S, et al. Phosphorylation of EIF2S1 (eukaryotic translation initiation factor 2 subunit alpha) is indispensable for nuclear translocation of TFEB and TFE3 during ER stress. Autophagy. 2023;19(7):2111–42. 10.1080/15548627.2023.2173900 PMC10283430 36719671

[14] Bhardwaj M, Leli NM, Koumenis C, Amaravadi RK (2020). Regulation of autophagy by canonical and non-canonical ER stress responses. Semin Cancer Biol.

[15] Rashid HO, Yadav RK, Kim HR, Chae HJ (2015). ER stress: Autophagy induction, inhibition and selection. Autophagy.

[16] Shi YH, Ding ZB, Zhou J, Hui B, Shi GM, Ke AW (2011). Targeting autophagy enhances sorafenib lethality for hepatocellular carcinoma via ER stress-related apoptosis. Autophagy.

[17] Ciechomska IA, Gabrusiewicz K, Szczepankiewicz AA, Kaminska B (2013). Endoplasmic reticulum stress triggers autophagy in malignant glioma cells undergoing cyclosporine a-induced cell death. Oncogene.

[18] Fromme JC, Orci L, Schekman R (2008). Coordination of COPII vesicle trafficking by Sec23. Trends Cell Biol.

[19] Jing J, Wang B, Liu P (2019). The functional role of SEC23 in vesicle transportation, autophagy and cancer. Int J Biol Sci.

[20] Lee H, Noh H, Mun J, Gu C, Sever S, Park S (2016). Anks1a regulates COPII-mediated anterograde transport of receptor tyrosine kinases critical for tumorigenesis. Nat Commun.

[21] Luo L, Wang Y, Du Y, Dong C, Ma A, Wang T (2020). MOG1 restores the expression and function of SCN5A-pR104W through sec23a-mediated forward trafficking. Eur Heart J.

[22] Chin A, Mariscal J, Kim M, Guerra G, Victor B, Qian C (2021). miR-1227 Targets SEC23A to regulate the shedding of large extracellular vesicles. Cancers (Basel).

[23] Sun Z, Liu D, Zeng B, Zhao Q, Li X, Chen H (2022). Sec23a inhibits the self-renewal of melanoma cancer stem cells via inactivation of ER-phagy. Cell Commun Signal.

[24] Zeng B, Zhao Q, Sun Z, Liu D, Chen H, Li X (2021). SEC23A Is an independent prognostic biomarker in bladder cancer correlated With MAPK signaling. Front Genet.

[25] Zeng B, Sun Z, Zhao Q, Liu D, Chen H, Li X (2021). SEC23A inhibit melanoma metastatic through secretory PF4 cooperation with SPARC to inhibit MAPK signaling pathway. Int J Biol Sci.

[26] Sun Z, Zeng B, Liu D, Zhao Q, Wang J, Rosie XH (2020). S100A8 transported by SEC23A inhibits metastatic colonization via autocrine activation of autophagy. Cell Death Dis.

[27] Luo Y, Zheng S, Wu Q, Wu J, Zhou R, Wang C (2021). Long noncoding RNA (lncRNA) EIF3J-DT induces chemoresistance of gastric cancer via autophagy activation. Autophagy.

[28] Hernandez I, Cohen M (2022). Linking cell-surface GRP78 to cancer: from basic research to clinical value of GRP78 antibodies. Cancer Lett.

[29] Wiseman RL, Mesgarzadeh JS, Hendershot LM (2022). Reshaping endoplasmic reticulum quality control through the unfolded protein response. Mol Cell.

[30] Sheshadri N, Poria DK, Sharan S, Hu Y, Yan C, Koparde VN (2021). PERK signaling through C/EBPdelta contributes to ER stress-induced expression of immunomodulatory and tumor promoting chemokines by cancer cells. Cell Death Dis.

[31] Wu S, Ye S, Lin X, Chen Y, Zhang Y, Jing Z (2021). Small hepatitis B virus surface antigen promotes malignant progression of hepatocellular carcinoma via endoplasmic reticulum stress-induced FGF19/JAK2/STAT3 signaling. Cancer Lett.

[32] Naama M, Telpaz S, Awad A, Ben-Simon S, Harshuk-Shabso S, Modilevsky S (2023). Autophagy controls mucus secretion from intestinal goblet cells by alleviating ER stress. Cell Host Microbe.

[33] Li LY, Chen XS, Wang KS, Guan YD, Ren XC, Cao DS (2020). RSK2 protects human breast cancer cells under endoplasmic reticulum stress through activating AMPKalpha2-mediated autophagy. Oncogene.

[34] Zhang HT, Zeng Q, Wu B, Lu J, Tong KL, Lin J (2021). TRIM21-regulated Annexin A2 plasma membrane trafficking facilitates osteosarcoma cell differentiation through the TFEB-mediated autophagy. Cell Death Dis.

[35] Morozova K, Sridhar S, Zolla V, Clement CC, Scharf B, Verzani Z (2015). Annexin A2 promotes phagophore assembly by enhancing Atg16L(+) vesicle biogenesis and homotypic fusion. Nat Commun.

[36] Huang Y, Jia M, Yang X, Han H, Hou G, Bi L (2022). Annexin A2: The diversity of pathological effects in tumorigenesis and immune response. Int J Cancer.

[37] Shalhout SZ, Yang PY, Grzelak EM, Nutsch K, Shao S, Zambaldo C (2021). YAP-dependent proliferation by a small molecule targeting annexin A2. Nat Chem Biol.

[38] Zhou B, Lu Q, Liu J, Fan L, Wang Y, Wei W (2019). Melatonin Increases the Sensitivity of Hepatocellular Carcinoma to Sorafenib through the PERK-ATF4-Beclin1 Pathway. Int J Biol Sci.

[39] Hill DS, Martin S, Armstrong JL, Flockhart R, Tonison JJ, Simpson DG (2009). Combining the endoplasmic reticulum stress-inducing agents bortezomib and fenretinide as a novel therapeutic strategy for metastatic melanoma. Clin Cancer Res.

[40] Lei Y, Henderson BR, Emmanuel C, Harnett PR, deFazio A (2015). Inhibition of ANKRD1 sensitizes human ovarian cancer cells to endoplasmic reticulum stress-induced apoptosis. Oncogene.

[41] Jeon YJ, Khelifa S, Ratnikov B, Scott DA, Feng Y, Parisi F (2015). Regulation of glutamine carrier proteins by RNF5 determines breast cancer response to ER stress-inducing chemotherapies. Cancer Cell.

[42] Chen J, Qiu M, Zhang S, Li B, Li D, Huang X (2022). A calcium phosphate drug carrier loading with 5-fluorouracil achieving a synergistic effect for pancreatic cancer therapy. J Colloid Interface Sci.

[43] Salaroglio IC, Panada E, Moiso E, Buondonno I, Provero P, Rubinstein M (2017). PERK induces resistance to cell death elicited by endoplasmic reticulum stress and chemotherapy. Mol Cancer.

[44] Blomme A, Peter C, Mui E, Rodriguez Blanco G, An N, Mason LM (2022). THEM6-mediated reprogramming of lipid metabolism supports treatment resistance in prostate cancer. EMBO Mol Med.

[45] Kiang KM, Tang W, Song Q, Liu J, Li N, Lam TL (2023). Targeting unfolded protein response using albumin-encapsulated nanoparticles attenuates temozolomide resistance in glioblastoma. Br J Cancer.

[46] Shi Y, Wang X, Meng Y, Ma J, Zhang Q, Shao G (2021). A Novel mechanism of endoplasmic reticulum stress- and c-Myc-degradation-mediated therapeutic benefits of antineurokinin-1 receptor drugs in colorectal cancer. Adv Sci (Weinh).

[47] Logue SE, McGrath EP, Cleary P, Greene S, Mnich K, Almanza A (2018). Inhibition of IRE1 RNase activity modulates the tumor cell secretome and enhances response to chemotherapy. Nat Commun.

[48] Song N, Song Y, Hu B, Liu X, Yu X, Zhou H (2023). Persistent endoplasmic reticulum stress stimulated by peptide assemblies for sensitizing cancer chemotherapy. Adv Healthc Mater.

[49] Gao Q, Li XX, Xu YM, Zhang JZ, Rong SD, Qin YQ (2020). IRE1alpha-targeting downregulates ABC transporters and overcomes drug resistance of colon cancer cells. Cancer Lett.

[50] Shi Z, Diao D, Zhao Y, Luo Y, Li Y, Liu D (2021). C/EBP homologous protein deficiency enhances hematopoietic stem cell function via reducing ATF3/ROS-induced cell apoptosis. Aging Cell.

[51] Pan Z, Zheng J, Zhang J, Lin J, Lai J, Lyu Z (2022). A Novel protein encoded by exosomal CircATG4B induces oxaliplatin resistance in colorectal cancer by promoting autophagy. Adv Sci (Weinh).

[52] Niu J, Yan T, Guo W, Wang W, Ren T, Huang Y, et al. The COPS3-FOXO3 positive feedback loop regulates autophagy to promote cisplatin resistance in osteosarcoma. Autophagy. 2023;19(6):1693–710. 10.1080/15548627.2022.2150003 PMC10262763 36451342

[53] Dong Y, Zhu G, Wang SF, Keon KA, Rubinstein JL, Zeng SX (2022). Toosendanin, a novel potent vacuolar-type H(+)-translocating ATPase inhibitor, sensitizes cancer cells to chemotherapy by blocking protective autophagy. Int J Biol Sci.

[54] Xu P, Xu S, Pan H, Dai C, Xu Y, Wang L (2023). Differential effects of the LncRNA RNF157-AS1 on epithelial ovarian cancer cells through suppression of DIRAS3- and ULK1-mediated autophagy. Cell Death Dis.

[55] Wang Y, Lieberman R, Pan J, Zhang Q, Du M, Zhang P (2016). miR-375 induces docetaxel resistance in prostate cancer by targeting SEC23A and YAP1. Mol Cancer.

[56] Zhaoran S, Linnebacher CS, Linnebacher M (2023). Increased SEC23A expression correlates with poor prognosis and immune infiltration in stomach adenocarcinoma. Cancers (Basel).

[57] Meng J, Liu K, Shao Y, Feng X, Ji Z, Chang B (2020). ID1 confers cancer cell chemoresistance through STAT3/ATF6-mediated induction of autophagy. Cell Death Dis.

