# Supplementary material for: SEC23A confers ER stress resistance in gastric cancer by forming the ER stress-SEC23A-autophagy negative feedback loop
Source: J Exp Clin Cancer Res. 2023 Sep 5;42:232. doi: 10.1186/s13046-023-02807-w (PMC10478313; doi:10.1186/s13046-023-02807-w)
Supplement: Supplementary file 1 — Additional file 1:Table S1. Sequences used for RNAi. Table S2. Primers used for qRT-PCR. Table S3. Antibodies used for western blotting. Table S4. Reagents used in the experiments. [file 13046_2023_2807_MOESM1_ESM.docx]

**Supplemental Tables**

Table S1. Sequences used for RNAi

| Name | Sequence (5^’^ to 3^’^) |
| --- | --- |
| shSEC23A-1 | GGAAGCTACAAGAATGGTTGT |
| shSEC23A-2 | GCACCTATTCTTACAGATGAT |
| siSTAT3 | GGGACCUGGUGUGAAUUAUTT |
| siATF6 | GGGACAUCAACAACCAAAUTT |
| siCHOP | GCCTGGTATGAGGACCTGCTT |
| siATF4 | GCCUAGGUCUCUUAGAUGATT |
| siCREB3L2 | GAGUCUUGUUCAACUGAGATT |
| siXBP1 | GTAAGAAATATTACTATAATT |

Table S2. Primers used for qRT-PCR

| Gene | Forward (5^’^ to 3^’^) | Reserves (5^’^ to 3^’^) |
| --- | --- | --- |
| SEC23A | CTTGCCGTGCAGTTTTGAATC | AGGACCACGCAGAACTACATATT |
| GAPDH | ATCATCAGCAATGCCTCCTG | ATGGACTGTGGTCATGAGTC |
| ATF6 | CGCCTTTTAGTCCGGTTCTT | CCAGTTGGTAACAATGCCATGT |
| CHOP | GGAAACAGAGTGGTCATTCCC | CTGCTTGAGCCGTTCATTCTC |
| ATF4 | CCCTTCACCTTCTTACAACCT | TGCCCAGCTCTAAACTAAAGGA |
| CREB3L2 | CACTGGGGTTGATTCCTCGTG | AATGCAGGTGGTCCACTGGG |
| STAT3 | CTGCCCCATACCTGAAGACC | TCCTCACATGGGGGAGGTAG |
| XBP1 | CCTTGTAGTTGAGAACCAGG | GGGGCTTGGTATATATGTGG |

Table S3. Antibodies used for western blotting

| Antibody | Company | Catalog number | Dilution ration |
| --- | --- | --- | --- |
| anti-SEC23A | abcam | ab137583 | 1:10000 |
| anti-BiP | proteintech | 11587-1-AP | 1:2000 |
| anti-GAPDH | proteintech | 60004-1-Ig | 1:100000 |
| anti-c-caspase3 | Cell Signaling Technology | 9664 | 1:1000 |
| anti-STAT3 | abcam | ab68153 | 1:1000 |
| anti-pY705-STAT3 | abcam | ab267373 | 1:1000 |
| anti-H3 | proteintech | 17168-1-AP | 1:2000 |
| anti-JAK2 | abcam | ab108596 | 1:5000 |
| anti-pY1007/1008-JAK2 | abcam | ab32101 | 1:5000 |
| anti-ATF6 | proteintech | 24168-1-AP | 1:2000 |
| anti-CHOP | proteintech | 15204-1-AP | 1:1000 |
| anti-ATF4 | proteintech | 10835-1-AP | 1:1000 |
| anti-XBP1 | protintech | 24868-1-AP | 1:1000 |
| anti-CREB3L2 | proteintech | 14514-1-AP | 1:1000 |
| anti-p62 | proteintech | 18420-1-AP | 1:10000 |
| anti-ATG5 | proteintech | 10181-2-AP | 1:1000 |
| anti-LC3B | Cell Signaling Technology | 3868 | 1:1000 |
| anti-ANXA2 | proteintech | 60051-1-Ig | 1:10000 |
| anti-TFEB | proteintech | 13372-1-AP | 1:1000 |
| anti-Na,K-ATPase A1 | Cell Signaling Technology | 23565 | 1:1000 |
| anti-c-PARP | abcam | ab32561 | 1:1000 |
| anti-Mouse IgG(H+L) | proteintech | SA00001-1 | 1:5000 |
| anti-Rabbit IgG(H+L) | Proteintech | SA00001-2 | 1:5000 |

Table S4. Reagents used in the experiments

| Reagents | Company | Catalog number |
| --- | --- | --- |
| DTT | Beyotime | ST041 |
| TM | MedChemExpress | HY-A0098 |
| 4-PBA | MedChemExpress | HY-A0281 |
| RAPA | MedChemExpress | HY-10219 |
| CQ | MedChemExpress | HY-17589A |
| AG490 | MedChemExpress | HY-12000 |
| S3I-201 | 10uM 24 h MedChemExpress | HY-15146 |
| 1. FU | Selleckchem | S1209 |
