# Supplementary material for: SEC23A confers ER stress resistance in gastric cancer by forming the ER stress-SEC23A-autophagy negative feedback loop
Source: J Exp Clin Cancer Res. 2023 Sep 5;42:232. doi: 10.1186/s13046-023-02807-w (PMC10478313; doi:10.1186/s13046-023-02807-w)
Supplement: Supplementary file 2 — Additional file 2: Figure S1. Additional results of Figure 2. (A) qRT-PCR (left) and western blotting (right) analysis of SEC23A expression in HGC27 cells under concentration gradient treatments of DTT for 12 h. (B) qRT-PCR (left) and western blotting (right) analysis of SEC23A expression in HGC27 cells under concentration gradient treatments of TM for 12 h. (C) qRT-PCR (upper) and western blotting (lower) analysis of SEC23A expression in HGC27 cells under concentration gradient treatments of H2O2 for 24 h. (D) qRT-PCR (upper) and western blotting (lower) analysis of SEC23A expression in HGC27 cells under concentration gradient treatments of 5-FU for 24 h. (E) Representative images of immunofluorescence co-staining SEC23A, BiP and cytokeratin in 15 GC paratumor tissues. (F) Fluorescence intensity quantitative analysis of SEC23A expression in BiP+ and BiP- areas of human GC paratumor tissues (n = 15). (G) Correlation between SEC23A and BiP fluorescent intensity in BiP+ areas of 15 paired GC and adjacent normal tissues. (n= 30). (H) Representative images of SEC23A and BiP western blotting results in 30 paired GC and adjacent normal tissues. (I) Quantitative analysis of SEC23A and BiP western blotting results in 30 paired GC and adjacent normal tissues. (J) Correlation between SEC23A and BiP protein expression in 30 paired GC and adjacent normal tissues. (n = 60). (K and L) Knockdown and overexpression efficiencies were verified in MKN45 and HGC27 cells by qRT-PCR (K) and western blotting (L) assays. (M and N) Western blotting assays to analysis the expression of BiP in the condition of SEC23A knockdown (M) or overexpression (N). Figure S2. Additional results of Figure. (A-E) Western blotting to analysis SEC23A expression changes in MKN45 and HGC27 cells under TM treatment (1.0 µg/ml, 12 h) after knockdown ATF4 (A), ATF6 (B), XBP1 (C), CHOP (D) and CREB3L2 (E) respectively. Figure S3. Additional results of Figure 4. (A) CCK 8 assays in MKN45 cells and HGC27 cells when [file 13046_2023_2807_MOESM2_ESM.docx]

**Supplementary figures**

**
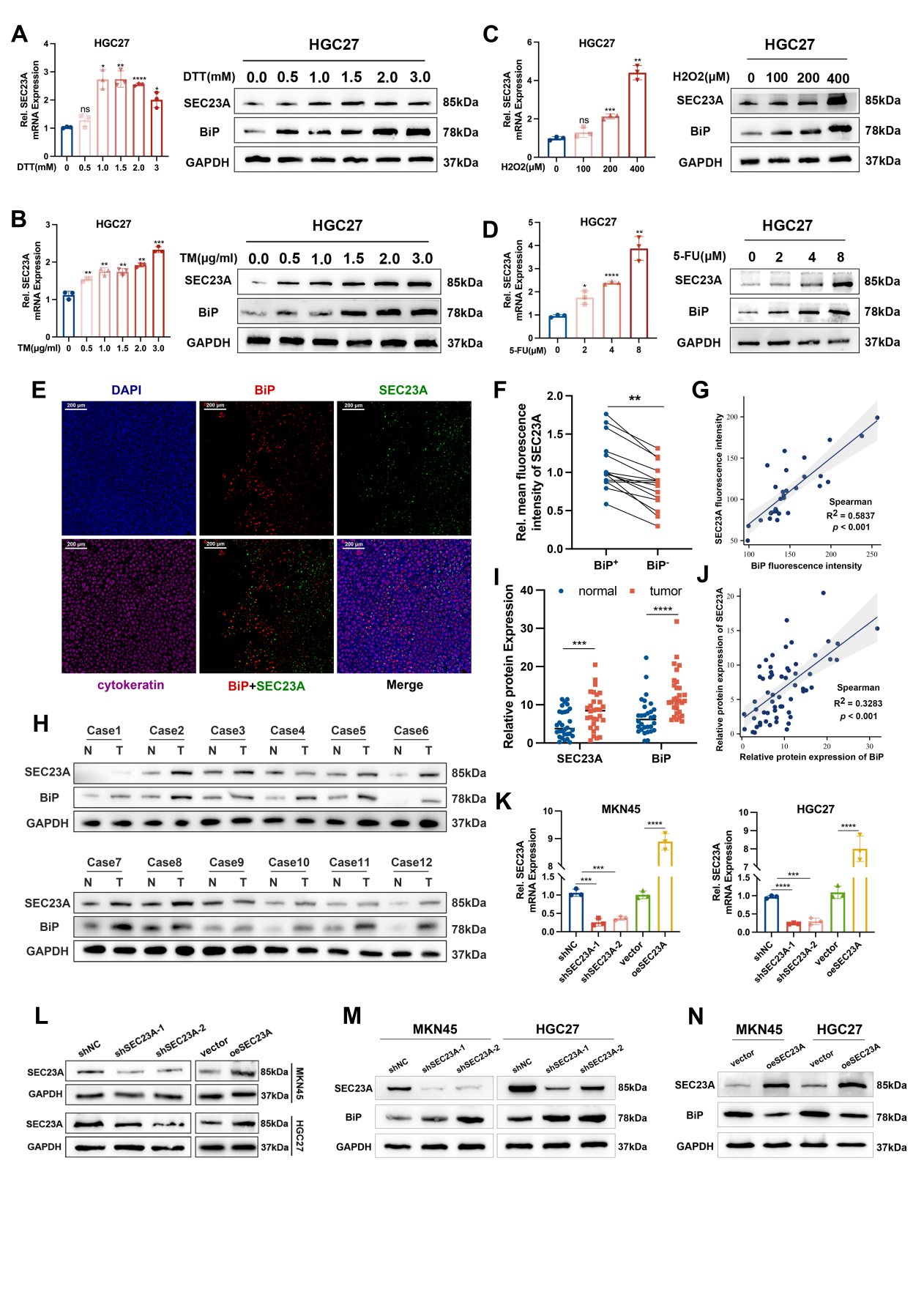
**

Figure S1. Additional results of Figure 2. (**A**) qRT-PCR (left) and western blotting (right) analysis of SEC23A expression in HGC27 cells under concentration gradient treatments of DTT for 12 h. (**B**) qRT-PCR (left) and western blotting (right) analysis of SEC23A expression in HGC27 cells under concentration gradient treatments of TM for 12 h. (**C**) qRT-PCR (upper) and western blotting (lower) analysis of SEC23A expression in HGC27 cells under concentration gradient treatments of H_2_O_2_ for 24 h. (D) qRT-PCR (upper) and western blotting (lower) analysis of SEC23A expression in HGC27 cells under concentration gradient treatments of 5-FU for 24 h. (**E**) Representative images of immunofluorescence co-staining SEC23A, BiP and cytokeratin in 15 GC paratumor tissues. (**F**) Fluorescence intensity quantitative analysis of SEC23A expression in BiP^+^ and BiP^-^ areas of human GC paratumor tissues (n = 15). (**G**) Correlation between SEC23A and BiP fluorescent intensity in BiP^+^ areas of 15 paired GC and adjacent normal tissues. (n = 30). (**H**) Representative images of SEC23A and BiP western blotting results in 30 paired GC and adjacent normal tissues. (**I**) Quantitative analysis of SEC23A and BiP western blotting results in 30 paired GC and adjacent normal tissues. (**J**) Correlation between SEC23A and BiP protein expression in 30 paired GC and adjacent normal tissues. (n = 60). (**K and L**) Knockdown and overexpression efficiencies were verified in MKN45 and HGC27 cells by qRT-PCR (**K**) and western blotting (**L**) assays. (**M and N**) Western blotting assays to analysis the expression of BiP in the condition of SEC23A knockdown (**M**) or overexpression (**N**).

**
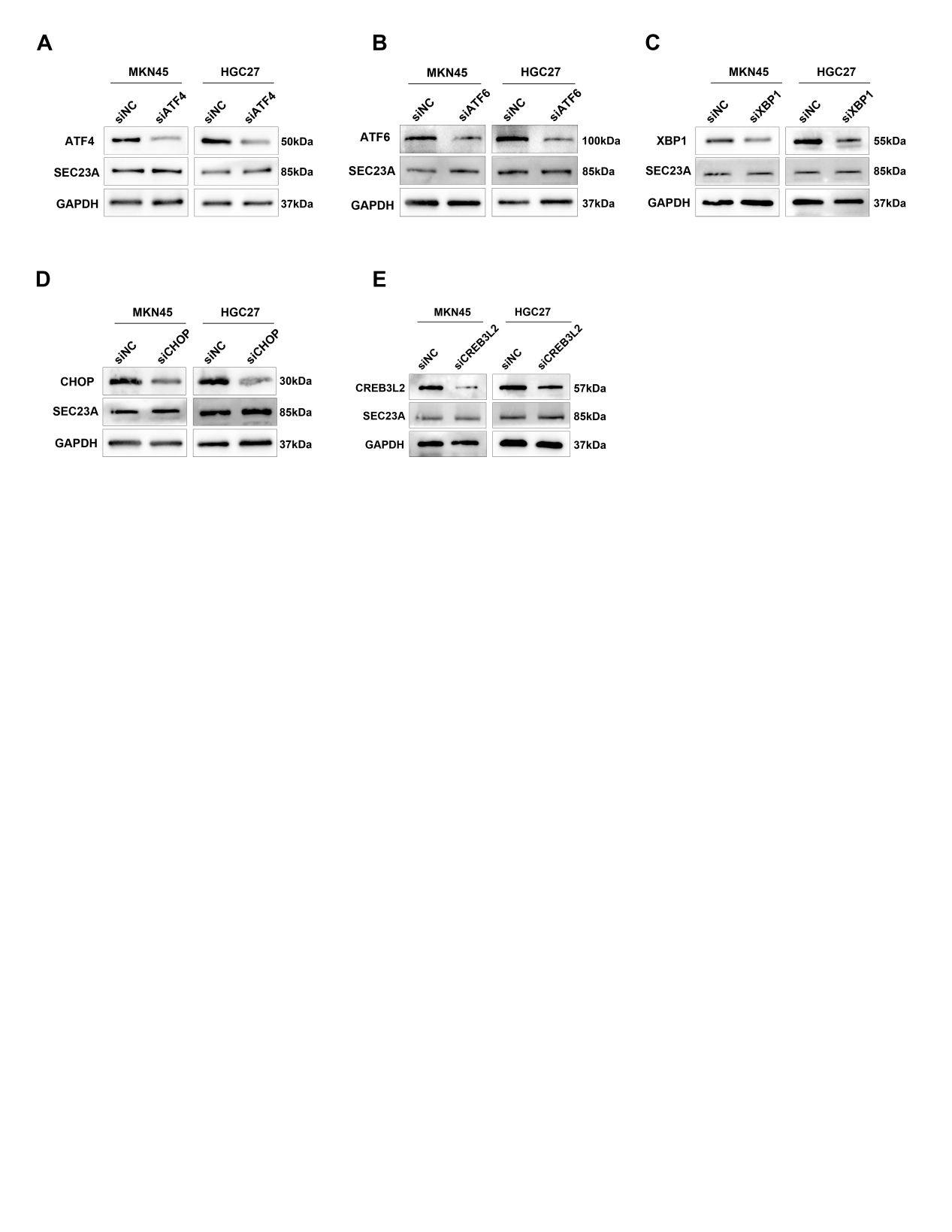
**

**Figure S2.** Additional results of Figure. (**A-E**) Western blotting to analysis SEC23A expression changes in MKN45 and HGC27 cells under TM treatment (1.0 μg/ml, 12 h) after knockdown ATF4 (**A**), ATF6 (**B**), XBP1 (**C**), CHOP (**D**) and CREB3L2 (**E**) respectively.

**
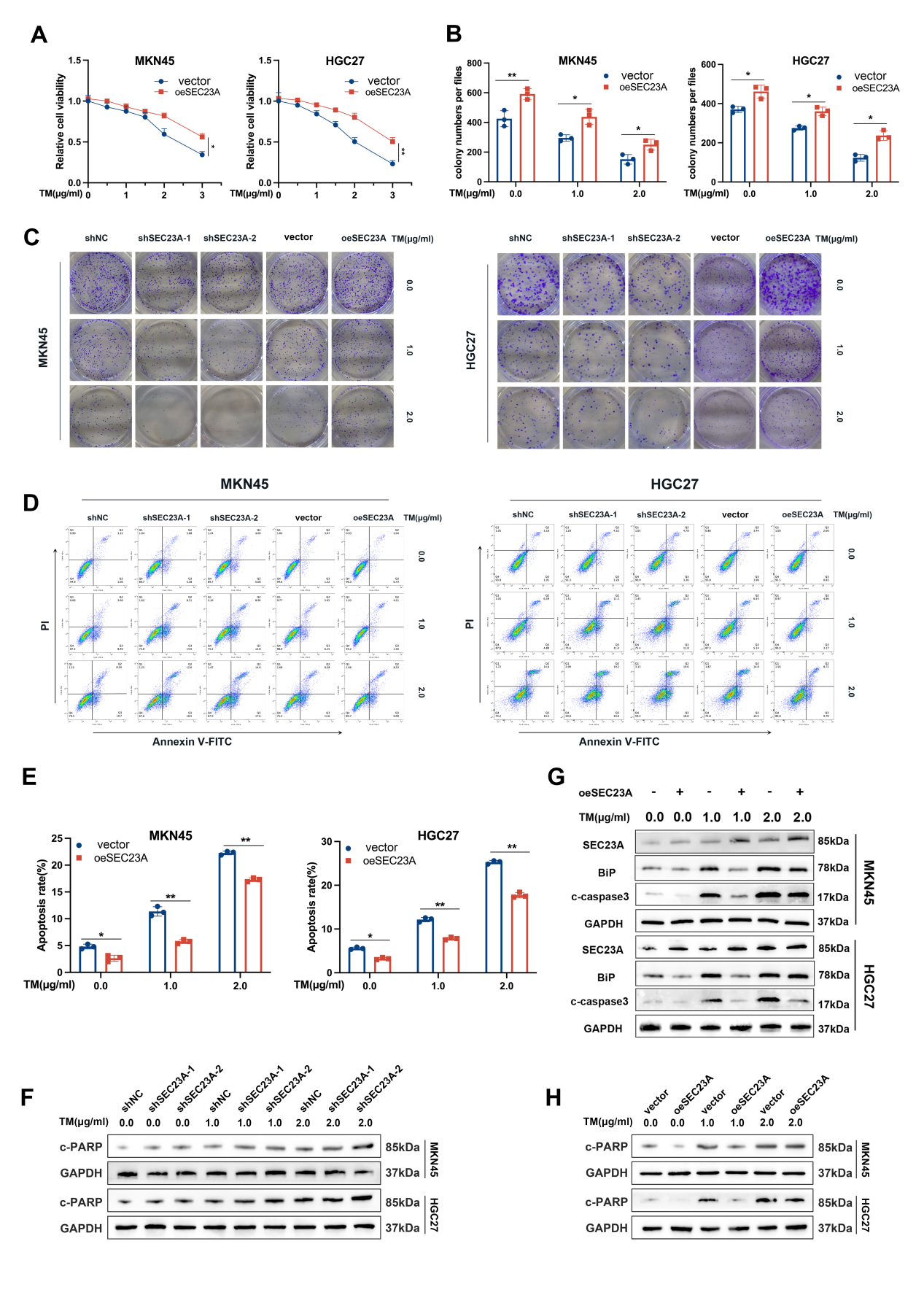
**

**Figure S3.** Additional results of Figure 4. (**A**) CCK 8 assays in MKN45 cells and HGC27 cells when SEC23A was overexpressed under concentration gradient TM treatments for 12 h. (**B**) Quantification of colony formation assays to determine cell survival in SEC23A overexpressed MKN45 and HGC27 cells treated with indicated doses of TM for 12 h. (**C and D**) Representative images of colony formation assays and flow cytometry in TM (indicated doses, 12 h) treated MKN45 and HGC27 cells with SEC23A knockdown, overexpression and corresponding control. (**E**) Quantification of flow cytometry to analysis cell apoptosis in SEC23A overexpressed MKN45 and HGC27 cells treated with indicated doses of TM for 12 h. (**F**) Western blotting against c-PARP performed on SEC23A silenced MKN45 and HGC27 cells under 12 h TM treatment with indicated doses. (**G and H**) Western blotting against c-caspase3 (**G**) and c-PARP (**H**) performed on SEC23A overexpressed MKN45 and HGC27 cells under 12 h TM treatment with indicated doses.

**
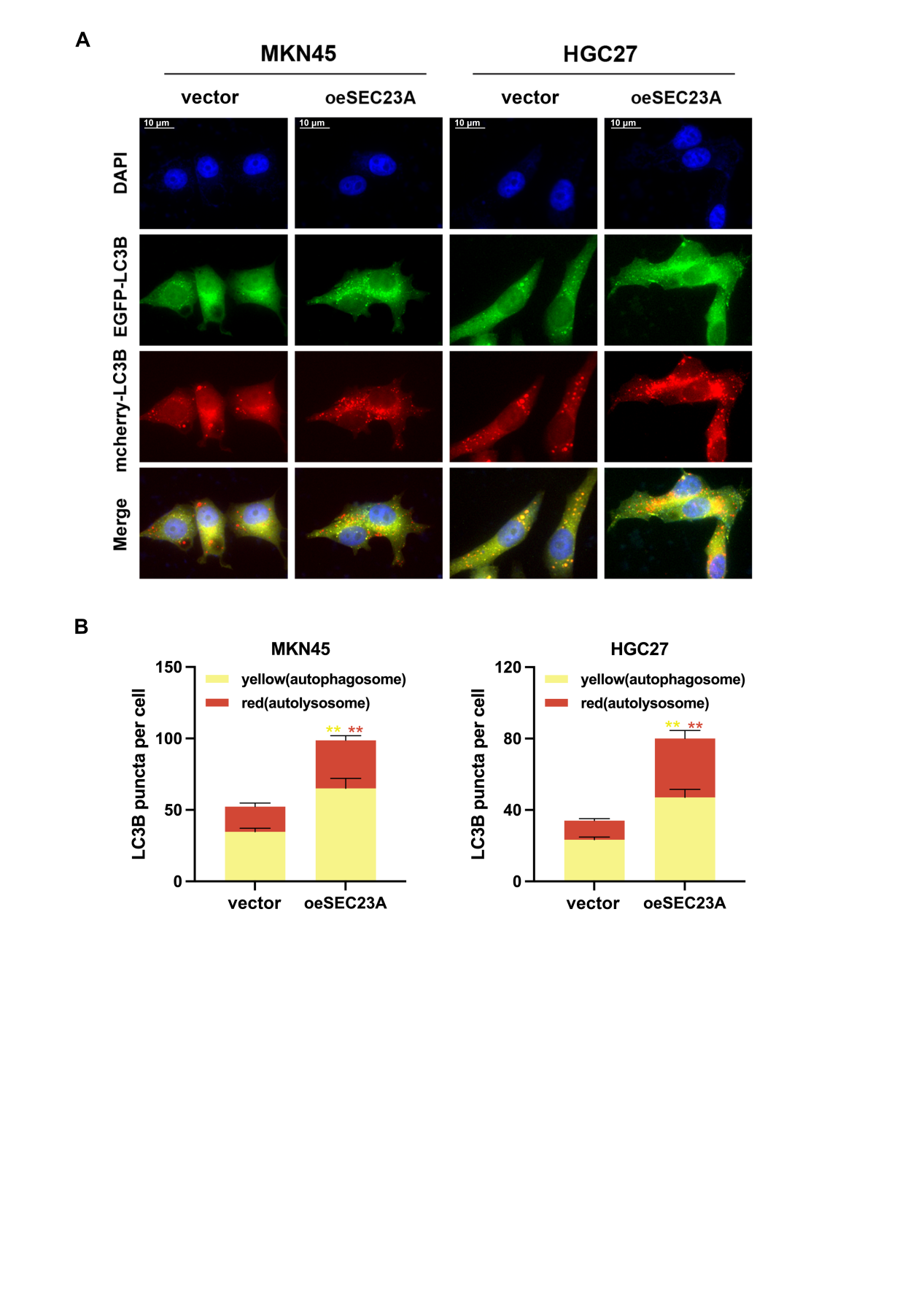
**

**Figure S4.** Additional results of Figure 5. (**A**) Representative immunofluorescence images of the mCherry-EGFP-LC3B transfected MKN45 and HGC27 cells with SEC23A overexpression and corresponding controls. (**B**) Quantification of the mCherry-EGFP-LC3B transfected MKN45 and HGC27 cells with SEC23A overexpression and corresponding controls.

**
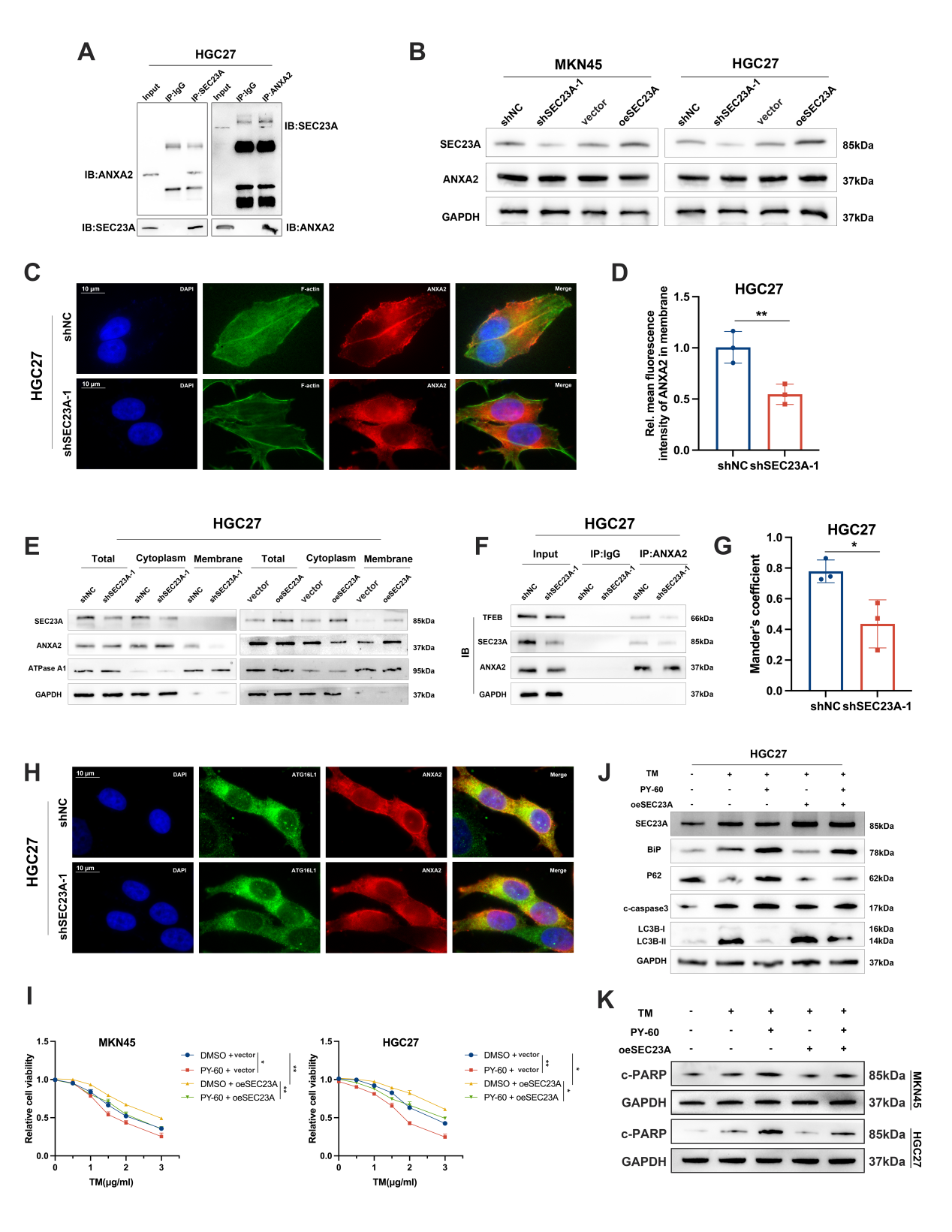
**

**Figure S5.** Additional results of Figure 6. (**A**) Co-IP assay using anti-SEC23A and anti-ANXA2 antibody to detect the binding of SEC23A and ANXA2 in HGC27 cells. (**B**) Western blotting assay to detect the expression of ANXA2 in MAN45 and HGC27 cells in the condition of SEC23A knockdown or expression. (**C and D**) Immunofluorescence images (**C**) and quantitation (**D**) to detect the cell membrane localization of ANXA2 in shNC and shSEC23A-1 HGC27 cells. (**E**) Western blotting analysis to detect the subcellular expression of ANXA2 in shNC and shSEC23A-1 HGC27 cells. (**F**) IP assay using anti-ANXA2 antibody to detect the content of TFEB bound to ANXA2 in shNC and shSEC23A-1 HGC27 cells. (**G and H**) Immunofluorescence quantitation (**G**) and images (**H**) to detect the co-localization of ANXA2 and ATG16L1 in shNC and shSEC23A-1 HGC27 cells. (**I**) CCK8 assays in MKN45 and HGC27 cells with indicated experimental settings. (**J**) Western blotting analysis to detect the expression of LC3B-I, LC3B-II, p62, BiP, c-caspase3 in HGC27 cells with indicated experimental settings. (**K**) Western blotting analysis to detect the expression of c-PARP in MKN45 and HGC27 cells with indicated experimental settings.

**
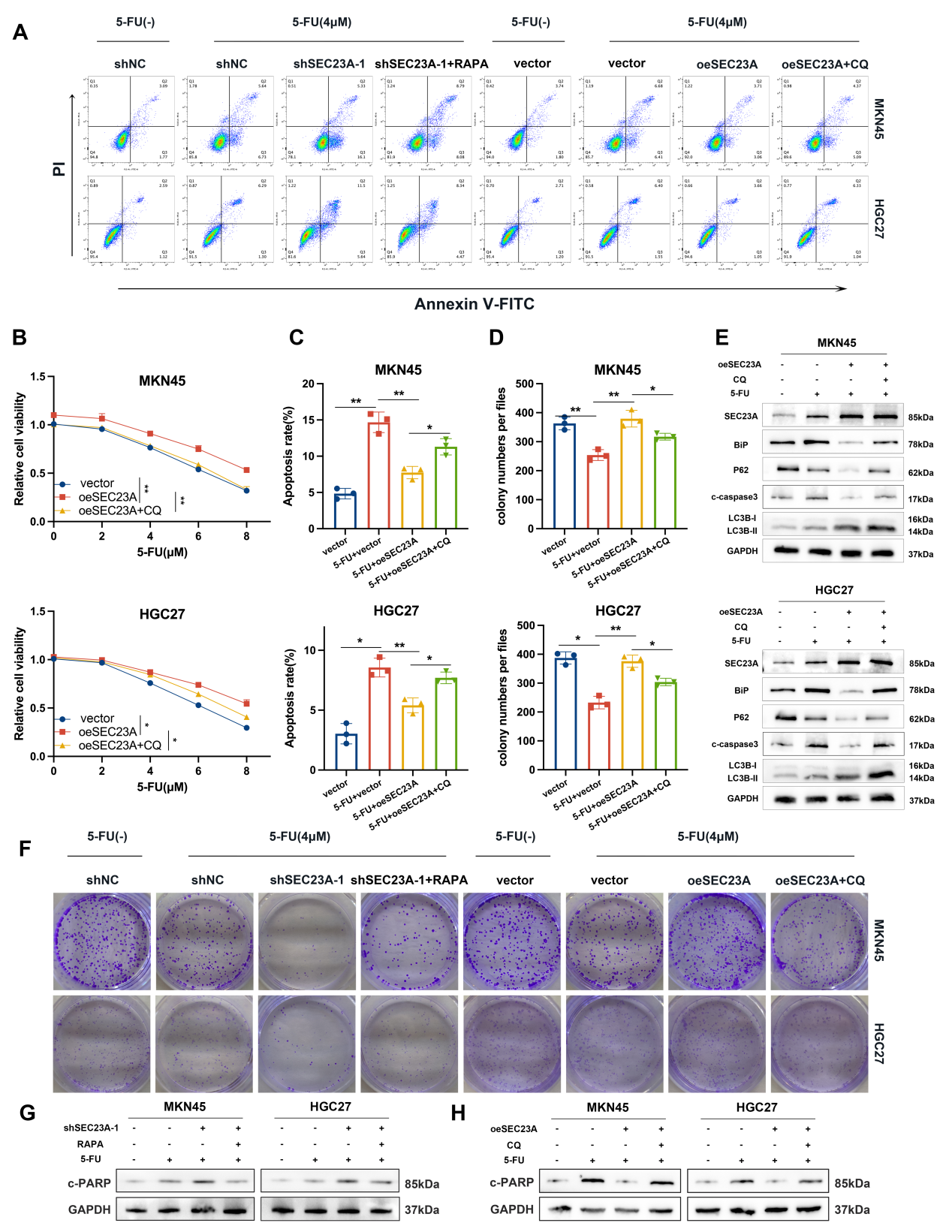
**

**Figure S6.** Additional results of Figure 8. (**A**) Representative images of flow cytometry of the indicated groups. (**B**) CCK8 assays of three groups (including vetor, oeSEC23A and oeSEC23A+CQ [10 μM, 12 h] ) in MKN45 cells and HGC27 cells under concentration gradient 5-FU for 12 h. (**C**) Flow cytometry quantification of the indicated groups in MKN45 and HGC27 cells with or without 4 μM 5-FU treatments for 12 h. (**D**) Colony formation assays quantification of the indicated groups in MKN45 and HGC27 cells with or without 4 μM 5-FU treatments for 12 h. (**E**) Western blotting against c-caspase3 and BiP in the indicated groups in MKN45 and HGC27 cells with or without 4 μM 5-FU treatments for 12 h. (**F**) Representative images of Colony formation assays of the indicated groups. (**G**) Western blotting against c-PARP in the indicated groups in MKN45 and HGC27 cells with or without 4 μM 5-FU treatments for 12 h
