# Supplementary material for: SEC23A confers ER stress resistance in gastric cancer by forming the ER stress-SEC23A-autophagy negative feedback loop
Source: J Exp Clin Cancer Res. 2023 Sep 5;42:232. doi: 10.1186/s13046-023-02807-w (PMC10478313; doi:10.1186/s13046-023-02807-w)
Supplement: Supplementary file 3 — Additional file 3. Supplemental Methods. [file 13046_2023_2807_MOESM3_ESM.docx]

**Supplemental Methods**

**Flow-cytometric apoptosis assays**

After indicated treatments, cells were digested by trypsin without EDTA, centrifuged at 1000 g for 5 min. Following washed with cold PBS, the cells were resuspended with Binding Buffer and stained with Annexin V and PI (Vazyme, A211) according to the kit protocols. The rate of apoptotic cells was detected by flow cytometry using the BD FACSCalibur system (BD Biosciences).

**Cell viability assay and Colony formation assay**

Cell viability was evaluated by Cell Counting Kit (CCK)-8 (Vazyme, A311). Cells were seeded at 2000 cells per well in 96-well plates and then incubated at 37°C for 24 h. After treatments, cells were incubated with CCK-8 solution for 2 h at 37°C and detected at the wavelength of 450 nm with microplate reader (Multiskan FC).

For the colony formation assay, cells were planted in six-well plates with 1000 cells per well. Then the cells were incubated with the indicated treatments 24 hours later. After continuously incubation at 37°C for 10–14 d, the cells were fixed with 4% paraformaldehyde for 15 min and stained with Crystal Violet Staining Solution (Beyotime, C0121) for 30 min.

**Immunofluorescence**

For cell immunofluorescence, cells were fixed in 4% paraformaldehyde for 10 min after corresponding treatments. Cell samples were permeabilized with Triton X-100 (Beyotime, P0096) for 5 min and blocked with Immunol Staining Blocking Buffer (Beyotime, P0102) for 1 h. After be treated with respective primary antibodies at

4°C overnight, cells were incubated with secondary antibodies at room temperature for 1 h, including Fluorescein (FITC)–conjugated Affinipure Goat Anti-Mouse IgG(H+L) (proteintech, SA00003-1, 1:100) and CoraLite647-conjugated AffiniPure F(ab')2 Fragment Goat Anti-Rabbit IgG (H+L) (proteintech, SA00014-9, 1:250). Nuclei were stained with DAPI (Beyotime, C1005).

For tissue immunofluorescence, after being incubated with corresponding primary antibodies, human GC slides were treated using a Four-color Fluorescence kit (Recordbio Biological Technology, RC0086-34R) based on the tyramide signal

amplification (TSA) technology according to the manufacture’s instruction.

These images were obtained by LEICA DMi8 system and analyzed by the ImageJ software. For each sample, quantitative analysis were based on four images taken at random fields.

The following primary antibodies were used for immunofluorescence: anti-cytokeratin (abcam, ab9377, 1:500); anti-SEC23A (abcam, ab137583, 1:500); anti-BiP (proteintech, 11587-1-AP, 1:250); anti-pY705-STAT3 (abcam, ab267373, 1:1000); anti-ATG16L1 (Cell Signaling Technology, 8089, 1:200); anti-ANXA2 (proteintech, 60051-1-Ig, 1:500).

**mCherry-EGFP-LC3B system**

Cells were transfected with Lenti-mCherry-EGFP-LC3B (Beyotime, C3002) to analysis autophagy flux. The mCherry-EGFP-LC3B transfected cells were incubated in confocal dish overnight and then treated with corresponding experimental settings. The cells were fixed with absolute ethyl alcohol for 20 min and then washed with PBS three times. Nuclei were stained with DAPI (Beyotime, C1005). These images were obtained by LEICA DMi8 system. Results were recorded as the number of yellow and red LC3B puncta in the merged images. The yellow and red fluorescence puncta represented autophagosomes and autolysosomes respectively.

**Subcellular fractionation assay**

Extraction of cytoplasmic, membrane and nuclear protein from cultured cells was preformed using Subcellular Protein Fractionation Kit (Thermo Fisher Scientific, 78840) according to the manufacturer’s instructions.

**Transmission electron microscopy (TEM)**

Collected cells was fixed with 2.5% glutaraldehyde at 4°C for 2 h and post-fixed with 1% osmium tetroxide in 0.1 M PB (pH 7.4) for 2 h at room temperature. Cells were dehydrated for 15 minutes respectively in successive concentrations of 50%, 70%, 80%, 90%, and 100% ethanol and 100% acetone. These samples were embedded and sectioned, then stained with 2% uranium acetate saturated alcohol solution avoiding light for 8 min and 2.6% lead citrate avoiding CO_2_ for 8 min, then dried at room temperature overnight. Finally, these samples were imaged by TEM (HITACHI, HT7800).

**Co-immunoprecipitation (Co-IP)**

The co-immunoprecipitation assay was preformed using the Immunoprecipitation Kit with Protein A+G Magnetic Beads (Beyotime, P2179) according to the kit's protocols. Primary antibodies used for co-immunoprecipitation assay included anti-SEC23A (Cell Signaling Technology, 8162, 1:50) and anti-ANXA2 (abcam, ab189473, 1:30). The co-immunoprecipitated proteins were detected by western blotting. SEC23A-binding proteins were analyzed using mass spectrometry.

**Chromatin immunoprecipitation (CHIP) assay**

Chromatin immunoprecipitation (ChIP) assays were performed using EZ-Magna ChIP™ A/G Chromatin Immunoprecipitation Kit (Merck millipore, 17-10086) according to instructions. Chromatin was crosslinked with 1% formaldehyde and then sheared to the size of 200–1000 bp by sonication. The primary antibody anti-pY705-STAT3 (abcam, ab267373) were used for CHIP assay. The ChIP-qPCR primers (forward: 5′-TGAGACCACCAAGCTCTAATGTC-3′; reverse: 5′-ATTGCCTCTGCCACTTCAACT-3′) were designed to amplify the promoter region containing the potential STAT3-binding site at the SEC23A promoter. The immunoprecipitated DNA samples were quantified by qRT–PCR and all results were normalized to the input.

**Immunohistochemistry (IHC)**

The human GC and [xenograft tumor](https://www.sciencedirect.com/topics/medicine-and-dentistry/tumor-xenograft) tissue sections were deparaffinized by xylene for 15 min thrice and soaked in 100%, 95%, 90%, 80%, and 70% ethyl alcohol for 5 min successively after dried at 60°C for 1 h. After antigen retrieval with Citrate-EDTA Antigen Retrieval Solution (Beyotime, P0086) at 100°C for 20 min, the slices were treated with 3% hydrogen peroxide and then blocked with 5% normal goat serum for 20 min at room temperature. They were respectively incubated with anti-SEC23A (abcam, ab137583, 1:400), anti-Ki67 (abcam, ab92742, 1:1000), anti-c-caspase3 (Cell Signaling Technology, 9664, 1:2000) and anti-BiP (proteintech, 11587-1-AP, 1:250) overnight at 4°C. The next day, IHC staining was performed with MaxvisionTM^2^ HRP-Polymer anti-Mouse/Rabbit IHC Kit (MXB Biotechnologies, KIT-5920) according to the instructions. They were imaged by Nikon microscopy. Immunostaining results of c-caspase3 were recorded as percentage of positive cell. Results of SEC23A and Ki67 were calculated with histochemistry score (H-Score, H-SCORE = ∑(pi×i) = percentage of weak intensity×1 + percentage of moderate intensity×2 + percentage of strong intensity×3).

**Tunel assay**

[Xenograft tumor](https://www.sciencedirect.com/topics/medicine-and-dentistry/tumor-xenograft) tissue sections were preformed Tunel assay with the TUNEL BrightRed Apoptosis Detection Kit (Vazyme, A113) according to the manufacturer's instructions. The images were acquired with a LEICA DMi8 system. Results were recorded as percentage of positive cell.

**Promoter luciferase reporter assay**

The three wide-type sequence containing predicted pY705-STAT3 binding sites (BS1, BS2 and BS3) and the mutant (BS2M) were established and cloned into the pGL-3 basic vector. These vectors were transfected with MKN45 cells. After the TM, S3I-201 and corresponding control treatments, the [luciferase](https://www.sciencedirect.com/topics/biochemistry-genetics-and-molecular-biology/luciferase) activity was detected on Dual-Luciferase system (Progema, Madison, USA) according to the manufacturer’ introduction. In addition, the reading mode for the dual luciferase detection was BottomRead. The [Renilla](https://www.sciencedirect.com/topics/pharmacology-toxicology-and-pharmaceutical-science/renilla)reporter system was applied to normalize the firefly luciferase.
