# Supplementary figures and images for: SEC23A confers ER stress resistance in gastric cancer by forming the ER stress-SEC23A-autophagy negative feedback loop
Source: J Exp Clin Cancer Res. 2023 Sep 5;42:232. doi: 10.1186/s13046-023-02807-w (PMC10478313; doi:10.1186/s13046-023-02807-w)

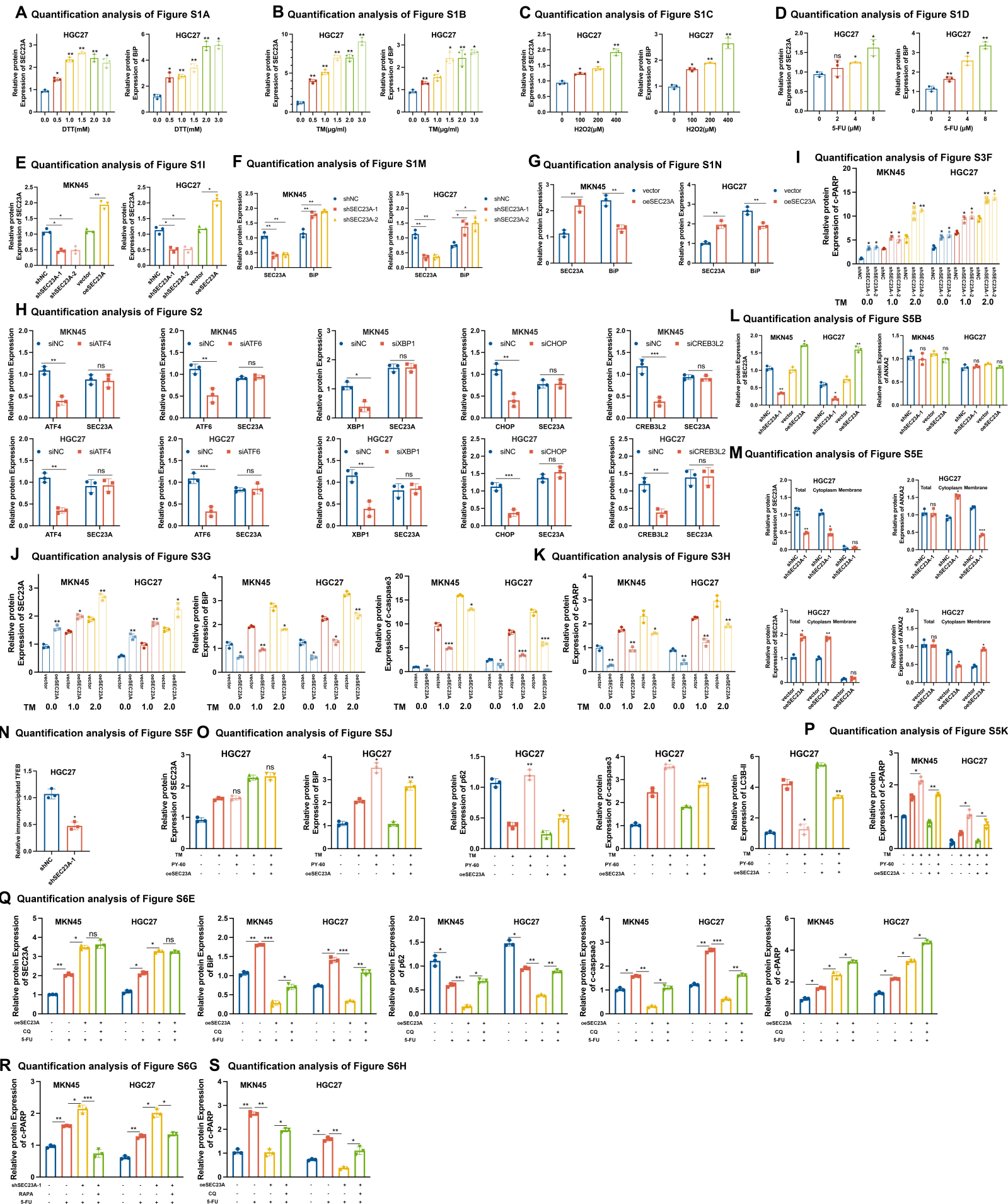

Supplement: Supplementary file 4 — Additional file 4. [file 13046_2023_2807_MOESM4_ESM.pdf]

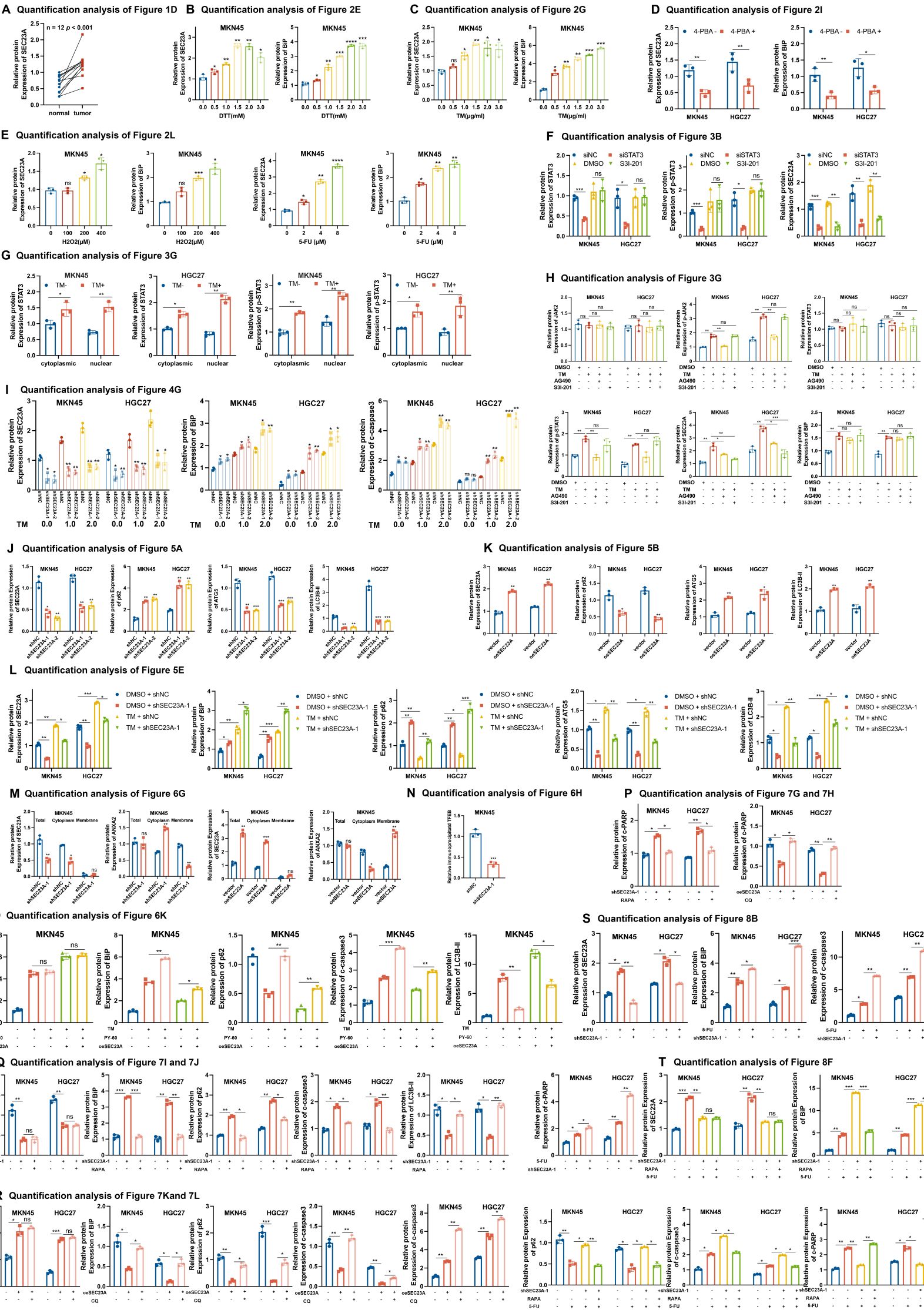

Supplement: Supplementary file 5 — Additional file 5. [file 13046_2023_2807_MOESM5_ESM.pdf]
